# Prevalence and predictability of the Chicago Classification of Pouchitis in ulcerative colitis: a multicenter study in Japan

**DOI:** 10.1007/s00535-025-02231-1

**Published:** 2025-03-06

**Authors:** Shintaro Akiyama, Ryohei Hayashi, Takeshi Takasago, Kurando Kusunoki, Hiroki Ikeuchi, Kento Takenaka, Kazuhiro Watanabe, Kazutaka Koganei, Nobuhiro Ueno, Mikihiro Fujiya, Naoki Hosoe, Fumikazu Koyama, Yasuhisa Sakata, Motohiro Esaki, Ken Takeuchi, Makoto Naganuma, Kiichiro Tsuchiya

**Affiliations:** 1 Department of Gastroenterology, Institute of Medicine, University of Tsukuba, 1-1-1 Tennodai, Tsukuba, Ibaraki 305-8575 Japan; 2 Department of Gastroenterology, Hiroshima University Hospital, Hiroshima, Japan; 3 Department of Gastroenterological Surgery, Division of Inflammatory Bowel Disease Surgery, Hyogo Medical University, Nishinomiya, Japan; 4 Department of Gastroenterology and Hepatology, Institute of Science Tokyo, Bunkyo-Ku, Tokyo, Japan; 5 Department of Surgery, Tohoku University Graduate School of Medicine, Sendai, Japan; 6 Department of Inflammatory Bowel Disease, Yokohama Municipal Citizen’s Hospital, Yokohama, Japan; 7 Division of Gastroenterology, Department of Internal Medicine, Asahikawa Medical University, Asahikawa, Japan; 8 Center for Preventive Medicine, School of Medicine, Keio University, Tokyo, Japan; 9 Department of Surgery, Nara Medical University, Kashihara, Japan; 10 Division of Gastroenterology, Department of Internal Medicine, Faculty of Medicine, Saga University, Saga, Japan; 11 Department of Gastroenterology, IBD Center, Tsujinaka Hospital Kashiwanoha, Chiba, Japan; 12 Division of Gastroenterology and Hepatology, Third Department of Internal Medicine, Kansai Medical University, Osaka, Japan

**Keywords:** Endoscopic phenotype, Chicago Classification, Chronic pouchitis, Pouch failure, Ulcerative colitis

## Abstract

**Background:**

Endoscopic phenotypes of pouchitis according to the Chicago Classification have been reported to be associated with poor pouch outcomes in ulcerative colitis (UC). Here, we aimed to assess the prevalence of endoscopic phenotypes and their predictability for pouch outcomes.

**Methods:**

This retrospective multicenter study included UC patients aged 18 years or older who underwent total colectomy between January 2000 and March 2020. The primary endpoints were frequencies of endoscopic phenotypes of the Chicago Classification and their predictability for chronic pouchitis and pouch failure. Endoscopic findings were evaluated at the initial pouchoscopy and at 3 and 10 years after ileostomy takedown.

**Results:**

A total of 392 eligible patients were identified. The frequencies of chronic pouchitis and pouch failure were 32% and 4.9%, respectively. Focal inflammation and inlet involvement at the initial postoperative pouchoscopy were significantly associated with subsequent risk of chronic pouchitis and pouch failure, respectively. Thirty percent of the patients with focal inflammation progressed to diffuse inflammation when chronic pouchitis developed. Multivariate analysis showed chronic pouchitis was significantly associated with diffuse inflammation and cuffitis observed throughout the clinical course. The proportion of pouch-related fistula was significantly lower in our cohort than in the US cohort (4.8% vs 19%, *P* < 0.001), and pouch-related fistula was an independent risk factor for pouch failure.

**Conclusions:**

We demonstrated the predictability of the Chicago Classification for pouch outcomes, and a lower prevalence of pouch-related fistula, resulting in a lower pouch failure risk in our multicenter cohort.

**Supplementary Information:**

The online version contains supplementary material available at 10.1007/s00535-025-02231-1.

## Introduction

Approximately 10% of patients with ulcerative colitis (UC) require total colectomy, with ileal pouch-anal anastomosis (IPAA) assuming the role of the rectum after surgery [1]. In up to 50% of those patients, acute pouchitis occurs, and 10% to 15% of patients with acute pouchitis develop chronic pouchitis [2, 3], thus requiring long-term antibiotic or biologic therapy. In a certain number of cases, pouch failure due to stenosis or fistula formation in the pouch occurs and requires diverting loop ileostomy (DLI) or pouch excision [4]. Appropriate monitoring of the IPAA is important to improve patients’ postoperative quality of life (QOL).

A previous study at the University of Chicago analyzed the endoscopic phenotype of pouchitis along with risk of pouch failure in patients with inflammatory bowel disease (IBD) and established the Chicago Classification of Pouchitis [5, 6]. This classification demonstrated that diffuse inflammation of the pouch body was significantly associated with risk of pouch excision [5]. A single-center retrospective study of UC in Japan showed that the endoscopic phenotypes occurred with the same frequencies as those of the UC patients in the Chicago Classification study [7]. Furthermore, long-term QOL was seriously impaired [8], and the risks of diverting ileostomy as well as chronic pouchitis were significantly increased by diffuse pouchitis of the Chicago Classification [7]. These results suggest that the Chicago Classification may be a universal classification to profile endoscopic pouch characteristics and to predict pouch outcomes in UC. However, the number of studies with an adequate sample size that have examined the frequency of endoscopic phenotypes and their associations with the risks of chronic pouchitis or pouch failure is still limited. Furthermore, which endoscopic phenotype at the initial postoperative pouchoscopy can predict pouch outcomes in UC remain unclear.

Therefore, we conducted a retrospective multicenter study to understand the prevalence of endoscopic phenotypes in our Japanese cohort and to clarify the clinical and endoscopic characteristics of patients with chronic pouchitis or pouch failure. Furthermore, we investigated the predictability of the Chicago Classification for these outcomes.

## Methods

### Eligible patients

This multicenter retrospective study was approved by the ethics committees of all 12 participating institutions in Japan (Table S1). The study was conducted in accordance with the Declaration of Helsinki. Since the study retrospectively analyzed existing clinical data and did not involve the collection of new samples, the requirement for informed consent was waived and the use of an opt-out consent approach was approved by the ethics committees. Eligibility criteria included patients with ulcerative colitis aged 18 years or older who underwent total colectomy for medical refractory disease or colorectal neoplasia between January 2000 and March 2020. The exclusion criteria were as follows: (1) patients with a preoperative diagnosis of Crohn disease (CD) or IBD-unclassified and (2) patients who had no pouchoscopies after ileostomy takedown.

### Endoscopic classification

Electronic data capture was used for data collection. The clinical data were extracted and analyzed by examining the electronic medical records of all the patients included in the study at each institution. Board-certified endoscopists reviewed endoscopic images and reports. Collaborating investigators at each participating hospital reviewed the endoscopic findings from their respective institutions. The endoscopic phenotype of the pouch was evaluated on the basis of the Chicago Classification [5] and classified as (1) normal, (2) afferent limb (AL) involvement, (3) inlet involvement, (4) diffuse inflammation, (5) focal inflammation of the pouch body, (6) cuffitis, or (7) pouch-related fistula that occurred after 6 months of stoma closure. Endoscopic findings were evaluated at the initial postoperative pouchoscopy and at 3 (± 1) years and 10 (± 1) years after the date of ileostomy takedown. If the initial pouchoscopies were performed at 3 years or 10 years after ileostomy takedown, these data were included in the initial postoperative pouchoscopy as well as in the data for each time point. Inflammatory findings on endoscopy based on the pouchitis disease activity index (PDAI) included erythema/edema, erosions/friability, ulceration, mucous exudates, stenosis, granularity, and loss of vascular pattern. [9, 10]

Endoscopic examinations with no evidence of inflammation at any anatomic location of the pouch were recorded as “normal pouch.” Endoscopic examinations with evidence of inflammation in the AL, inlet, or rectal cuff were recorded as “AL involvement,” “inlet involvement,” or “cuffitis,” respectively. “Pouchitis” was defined as one or more inflammatory findings in the tip, proximal, or distal areas of the pouch. Two or more endoscopic findings in all the anatomic locations of the pouch body (tip, proximal, and distal pouch) were defined as “diffuse inflammation of the pouch body.” Cases of pouchitis that did not meet the criteria for diffuse inflammation were recorded as “focal inflammation of the pouch body.” [5] “Pouch-related fistula” was defined as any type of fistula noted on endoscopy or other imaging studies ≥ 6 months after ileostomy takedown. CD-like pouch inflammation (CDLPI) was also defined as a pouch with AL involvement, stenosis at any anatomic location of the pouch, or pouch-related fistula [11]. The number of inflammatory phenotypes was determined as the number of phenotypes other than the normal phenotype.

In this study, the initial and overall endoscopic phenotypes were assessed in each patient. The initial phenotype was determined on the basis of the first pouchoscopy performed after ileostomy takedown. Endoscopic examinations at all time points were evaluated to determine the overall phenotype, which reflects the phenotypes during the entire clinical course. If all endoscopic examinations in an individual patient were reported as normal, the patient was categorized into the “normal” phenotype as an overall phenotype. If an inflammatory phenotype was identified on at least one endoscopic examination, the patient was included in the analysis for the respective phenotypic category. The finding of focal inflammation and diffuse inflammation of the pouch body on separate endoscopic examinations was recorded as “diffuse inflammation of the pouch body” rather than as focal inflammation of the pouch body as an overall phenotype.

### Methods for evaluating endpoints

The primary endpoints were the prevalence of endoscopic phenotypes and their predictability and association with pouch outcome. The frequency of phenotypes was compared with the published data of 382 UC patients with IPAA who underwent pouchoscopy at the University of Chicago between June 1997 and December 2019. [6] Pouch outcomes included chronic pouchitis and pouch failure. Chronic pouchitis was defined as a condition in which the clinical symptoms (e.g., stool frequency, rectal bleeding, fecal urgency or abdominal cramps, and fever) [9, 10] persisted for more than 4 weeks despite antibiotic therapy, thus requiring long-term antibiotic and anti-inflammatory therapies. A pouch condition in which antibiotics can be temporarily discontinued but relapse after several months was also included as chronic pouchitis. Pouch failure was defined as a condition in which the pouch required DLI or pouch excision.

To clarify which initial phenotype could predict the pouch outcomes, Kaplan–Meier (KM) curves were described from the date of the initial postoperative pouchoscopy showing each phenotype to the date of pouch outcomes. Survival estimates were compared by the log-rank test. Data were censored at the date of the patient’s last visit. Patients who had outcomes before the initial postoperative pouchoscopy were excluded. To determine the predictability and association of clinical factors and endoscopic phenotype with each pouch outcome, multivariate analysis was performed with the Cox proportional hazards model and a logistic regression model, respectively, including the variables identified in the univariate analysis (*P* < 0.10; variables with the smallest *P*-values were superiorly selected) that were not associated with each other.

The secondary endpoints were initial and overall endoscopic phenotype of acute pouchitis. Acute pouchitis was defined as a condition in which clinical symptoms responded to 2 weeks of antibiotics and resolved within 4 weeks. In addition, the correlation between the endoscopic phenotype and clinical subscore of the PDAI on the day of pouchoscopy or the most recent clinical visit was also assessed to understand the influence of the endoscopic phenotype on pouchitis-related symptoms.

All statistical analyses were performed using R (version 4.2.1). *P*-values of less than 0.05 were considered significant.

## Results

### Patient characteristics

In all, 465 patients were enrolled. After screening and eligibility assessment, 392 UC patients were included (Fig. S1). The median observation period from the date of ileostomy takedown was 8.5 years (IQR, 4.8–12.7). The patient characteristics are shown in Table 1.

**Table 1:** Patient characteristics

Characteristic	*N*^1^	
Age at diagnosis (yrs)	389	32 (22, 45)
Age at colectomy (yrs)	392	44 (31, 54)
Disease duration until surgery (yrs)	389	5 (2, 13)
Body mass index	391	20.4 (18.4, 23.0)
Gender	392	
Female		168 (43%)
Male		224 (57%)
Montreal classification	379	
Proctitis		6 (1.6%)
Left-sided colitis		66 (17%)
Extensive colitis		307 (81%)
Primary sclerosing cholangitis	390	
No		382 (98%)
Yes		8 (2.1%)
Current smoker	286	
No		275 (96%)
Yes		11 (3.8%)
Stage of ileal pouch-anal anastomosis	386	
1-stage		55 (14%)
2-stages		232 (60%)
3-stage		99 (26%)
Anastomosis type	376	
Staple (IACA; ileoanal canal anastomosis)		157 (42%)
Hand-sewn (IAA; ileoanal anastomosis)		219 (58%)
Preoperative treatments		
Tumor necrosis factor inhibitors	373	116 (31%)
Azathioprine/6-mercaptopurine	373	166 (45%)
Ustekinumab	373	0 (0%)
Vedolizumab	373	4 (1.1%)
Janus kinase inhibitors	373	12 (3.2%)
Oral aminosalicylates	373	324 (87%)
Calcineurin inhibitors	373	111 (30%)
Systemic steroids	373	335 (90%)
Apheresis	373	168 (45%)
No preoperative treatments	373	2 (0.5%)
Indications for colectomy		
Medically refractory	389	266 (68%)
Dysplasia/Colorectal Cancer	389	85 (22%)
Fulminant colitis	389	6 (1.5%)
Toxic megacolon	389	13 (3.3%)
Massive hemorrhage	389	14 (3.6%)
Perforation	389	15 (3.9%)
Postoperative complications		
No postoperative complications	376	214 (57%)
Anastomosis leak	376	22 (5.9%)
Pelvic sepsis	376	6 (1.6%)
Abdominal abscess requiring drainage	376	19 (5.1%)
Ileus	376	89 (24%)
Fistulas or sinus tracts developed until ileostomy takedown	376	7 (1.9%)
Postoperative treatments		
Loperamide	390	282 (72%)
Metronidazole/ciprofloxacin	390	219 (56%)
Oral aminosalicylates	390	44 (11%)
Topical aminosalicylates	390	36 (9.2%)
Oral steroids	390	32 (8.2%)
Topical steroids	390	50 (13%)
Tumor necrosis factor inhibitors	390	22 (5.6%)
Azathioprine/6-mercaptopurine	390	8 (2.1%)
Ustekinumab	390	3 (0.8%)
Vedolizumab	390	8 (2.1%)
Janus kinase inhibitors	390	3 (0.8%)
Calcineurin inhibitors	390	2 (0.5%)
No postoperative treatments	390	23 (5.9%)
Duration between last surgery and 1st scope (yrs)	392	1.02 (0.54, 2.25)
Follow-up (yrs)	385	8.5 (4.8, 12.7)

^1^Median (IQR); *n* (%)

### Prevalence of endoscopic phenotype of the Chicago Classification

The number of pouchoscopies was 392 at the initial postoperative endoscopy, and 258 at 3 years and 99 at 10 years after the ileostomy takedown. The median time between the date of ileostomy takedown and the date of the initial postoperative pouchoscopy was 1.0 year (IQR, 0.54–2.3). The most common initial phenotype observed at the first postoperative endoscopy was focal inflammation of the pouch body (59%), followed by cuffitis (50%), inlet involvement (34%), AL involvement (20%), normal phenotype (20%), diffuse inflammation (16%), and pouch-related fistula (1.0%) (Table 2).

**Table 2:** Pouch outcomes and endoscopic phenotypes

Characteristic	*N*^1^	
Pouch outcomes		
Chronic pouchitis	390	125 (32%)
Acute pouchitis	377	115 (31%)
Diverting loop ileostomy	389	19 (4.9%)
Pouch excision	387	3 (0.8%)
Pouch failure	389	19 (4.9%)
Phenotypes at the initial postoperative scope		
Normal	392	80 (20%)
Afferent limb involvement	280	57 (20%)
Inlet involvement	325	112 (34%)
Diffuse inflammation of the pouch body	390	63 (16%)
Focal inflammation of the pouch body	390	230 (59%)
Cuffitis	250	125 (50%)
Pouch-related fistula	392	4 (1.0%)
Number of inflammatory phenotypes at the first scope	392	
0		80 (20%)
1		122 (31%)
2–3		168 (43%)
4–5		22 (5.6%)
Phenotypes at 3 years after ileostomy takedown		
Normal (3yrs)	258	55 (21%)
Afferent limb involvement (3yrs)	174	38 (22%)
Inlet involvement (3yrs)	204	70 (34%)
Diffuse inflammation of the pouch body (3yrs)	258	49 (19%)
Focal inflammation of the pouch body (3yrs)	258	143 (55%)
Cuffitis (3yrs)	177	96 (54%)
Pouch-related fistula (3yrs)	256	7 (2.7%)
Number of inflammatory phenotypes at 3yrs	258	
0		55 (21%)
1		73 (28%)
2–3		109 (42%)
4–5		21 (8.1%)
Phenotypes at 10 years after ileostomy takedown		
Normal (10yrs)	99	12 (12%)
Afferent limb involvement (10yrs)	70	18 (26%)
Inlet involvement (10yrs)	75	37 (49%)
Diffuse inflammation of the pouch body (10yrs)	99	11 (11%)
Focal inflammation of the pouch body (10yrs)	99	75 (76%)
Cuffitis (10yrs)	83	48 (58%)
Pouch-related fistula (10yrs)	99	6 (6.1%)
Number of inflammatory phenotypes at 10yrs	99	
0		12 (12%)
1		16 (16%)
2–3		61 (62%)
4–5		10 (10%)
Overall phenotypes determined by all scopes		
Normal (Overall)	392	57 (15%)
Afferent limb involvement (Overall)	336	88 (26%)
Inlet involvement (Overall)	363	168 (46%)
Diffuse inflammation of the pouch body (Overall)	390	106 (27%)
Focal inflammation of the pouch body (Overall)	390	216 (55%)
Cuffitis (Overall)	265	155 (58%)
Pouch-related fistula (Overall)	392	19 (4.8%)
Number of overall inflammatory phenotypes	392	
0		57 (15%)
1		95 (24%)
2–3		188 (48%)
4–5		52 (13%)

^ 1^*n* (%)

Meanwhile, the most common overall endoscopic phenotype, which was determined by postoperative endoscopic examinations at all time points, was cuffitis (58%), followed by focal inflammation (55%), inlet involvement (46%), diffuse inflammation (27%), AL involvement (26%), normal phenotype (15%), and pouch-related fistula (4.8%) (Table 2). The most common type of fistula was perianal fistula, and the most common treatment was antibiotics (Table S2). We also evaluated the postoperative management of patients with CDLPI, defined as a pouch with AL involvement, stenosis at any anatomic location of the pouch, or pouch-related fistula [11]. We identified 110 patients with CDLPI, who were more likely to be treated with antibiotics and tumor necrosis factor inhibitors. Furthermore, the risk of diverting stoma and pouch excision was significantly higher in patients with CDLPI compared to those without CDLPI (Table S3), consistent with previous studies. [12]

**Fig. 1:**
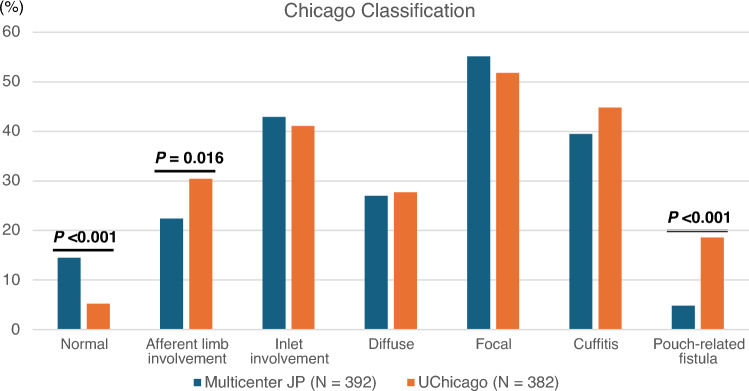
Prevalence of endoscopic phenotypes according to the Chicago Classification. The prevalence of phenotypes was compared with the published data of 382 UC patients from the University of Chicago. To compare the phenotype data between our multicenter cohort and the University of Chicago cohort, the denominator for phenotype prevalence was the number of overall patients in this analysis

Comparison of patient characteristics between our cohort and the University of Chicago cohort [6] showed that the percentage of patients undergoing 3-stage IPAA was significantly higher in the University of Chicago cohort than in our cohort, whereas the rate of colorectal neoplasia and hand-sewn anastomosis was significantly higher in our cohort than in their cohort. Additionally, pre- and post-operative data showed that patients with UC at the University of Chicago were more frequently treated with immunosuppressive therapies and were more likely to experience postoperative complications and pouch excision compared to our cohort (Table S4). Endoscopic phenotype data from the University of Chicago [6] showed that the proportion of normal phenotype was significantly higher in our cohort than in the University of Chicago cohort. Notably, the frequencies of pouch-related fistula and AL involvement were significantly lower in our cohort (4.8% and 22.4%, respectively) compared to the University of Chicago [6] (18.6% and 30.4%, respectively). The frequencies of the other phenotypes were comparable between the two cohorts (Fig. 1, Table S4).

### Predictability of initial phenotypes and contributing factors for chronic pouchitis

**Fig. 2:**
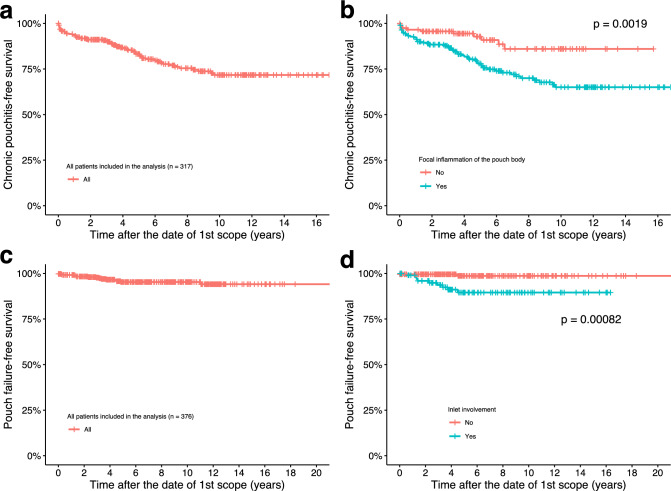
Kaplan–Meier curves evaluating chronic pouchitis-free survival for** a** overall population and** b** focal inflammation of the pouch body observed at the initial postoperative pouchoscopy. Kaplan–Meier curves evaluating pouch survival for** c** overall population and **d** inlet involvement observed at the initial postoperative pouchoscopy

The frequency of chronic pouchitis was 32% (Table 2). A KM curve showed that the 10-year chronic pouchitis-free survival rate was 71.7% (95% CI 65.3–78.7%) (Fig. 2a).

We examined which initial phenotype in the first postoperative scope was associated with subsequent risk of chronic pouchitis (*n* = 317). This examination showed that patients with an initial phenotype of focal inflammation had a significantly higher risk of chronic pouchitis over time (*P* = 0.0019), whereas patients with the normal phenotype as an initial phenotype had a significantly lower risk of chronic pouchitis (*P* = 0.0035) (Fig. 2b, Fig. S2). The same significant results were obtained even in asymptomatic patients (PDAI clinical subscore 0, *n* = 119) at the initial pouchoscopy (Fig. S3). The Cox proportional hazards model including the 6 factors with the smallest *P*-values (Table S5) showed that an initial phenotype of focal inflammation was a significant predictor of chronic pouchitis (HR, 2.2; 95% CI 1.1–4.4;* P* = 0.033) (Table 3). Hand-sewn anastomosis was inversely associated with the risk of chronic pouchitis (HR, 0.55; 95% CI 0.31–0.98;* P* = 0.042) (Table 3), indicating that the residual rectal cuff resulting from stapled anastomosis may be associated with chronic pouchitis.

**Table 3:** Cox proportional hazards model to assess factors predicting pouch outcomes

Characteristic	HR^1^	95% CI^1^	*P*-value*
Chronic pouchitis			
Age at colectomy (yrs)	0.96506	0.94463, 0.98593	**0.00112**
Anastomosis type			
1. Staple	–	–	
2. Hand-sewn	0.55299	0.31227, 0.97927	**0.04217**
Focal inflammation of the pouch body at the initial postoperative scope	2.15214	1.06298, 4.35727	**0.03320**
Preoperative azathioprine/6-mercaptopurine	1.28913	0.75895, 2.18966	0.34744
Dysplasia/Colorectal Cancer	0.96549	0.34947, 2.66741	0.94600
Disease duration until surgery (yrs)	1.00014	0.95737, 1.04482	0.99504
Pouch failure			
Age at colectomy (yrs)	0.92806	0.86834, 0.99190	**0.02783**
No postoperative complications	0.59421	0.13932, 2.53438	0.48183
Preoperative tumor necrosis factor inhibitors	4.55330	1.07511, 19.2841	**0.03956**
Inlet involvement at the initial postoperative scope	6.31348	1.29217, 30.8474	**0.02281**

^1^*HR*   Hazard Ratio, *CI* Confidence Interval

^*^The bold values indicate *p-*value < 0.05

To understand which endoscopic items increase the risk of chronic pouchitis, we performed a subgroup analysis of patients with an initial phenotype of focal inflammation and showed that patients with chronic pouchitis were more likely to have erythema/edema at the distal pouch body than were patients without chronic pouchitis (Table S6 and Fig. S4), although not significant (*P* = 0.083). Whilst the inflammatory phenotypes and their number in the initial pouchoscopy did not differ, the number of inflammatory phenotypes as well as the percentages of diffuse inflammation, cuffitis, and pouch-related fistula as overall phenotypes were significantly higher in patients with chronic pouchitis than in those without chronic pouchitis. Notably, 30% of patients who later developed chronic pouchitis had a progression from focal to diffuse inflammation of the pouch body (Table S6). Images of a representative case are shown in Fig. S5.

Next, we analyzed the contributing factors to chronic pouchitis. To evaluate the phenotype that developed during the entire clinical course, the overall phenotype was included in this analysis. The rates of AL involvement, inlet involvement, diffuse inflammation of the pouch body, cuffitis, and pouch-related fistula as overall phenotypes were significantly higher in patients with chronic pouchitis than in those without it (Table S7). On logistic regression analysis, chronic pouchitis was significantly associated with diffuse inflammation of the pouch body (OR, 3.8; 95% CI 1.4–10.5; *P* = 0.009) and cuffitis (OR, 2.9; 95% CI 1.3–7.3; *P* = 0.015) as the overall endoscopic phenotype. Conversely, age at colectomy was inversely associated with risk of chronic pouchitis (OR, 0.97; 95% CI 0.943–0.996; *P* = 0.028) (Table 4).

**Table 4:** Logistic regression analysis to assess factors contributing to pouch outcomes

Characteristic	OR^1^	95% CI^1^	*p*-value*
Chronic pouchitis			
Age at colectomy (yrs)	0.96970	0.94268, 0.99602	**0.02758**
Preoperative systemic steroids	2.49855	0.34919, 51.3805	0.42985
Medically refractory disease	1.00661	0.37317, 2.83359	0.98974
Abdominal abscess requiring drainage	0.00000	Not estimable	0.99013
Afferent limb involvement (Overall)	1.04546	0.36992, 2.84891	0.93143
Inlet involvement (Overall)	1.78436	0.64629, 4.80613	0.25491
Diffuse inflammation of the pouch body (Overall)	3.76542	1.42434, 10.4575	**0.00870**
Cuffitis (Overall)	2.94634	1.25774, 7.30114	**0.01520**
Pouch-related fistula (Overall)	1.88641	0.37187, 11.0455	0.45132
Pouch failure*			
Afferent limb involvement (Overall)	3.17363	0.70575, 18.2191	0.15797
Inlet involvement (Overall)	2.11203	0.28059, 19.6069	0.47542
Cuffitis (Overall)	2.22275	0.43606, 17.3235	0.37572
Pouch-related fistula (Overall)	13.8496	3.00998, 67.8603	**0.00075**

^1^*OR*  Odds Ratio, *CI*   Confidence Interval

^*^Since the number of events was small, only endoscopic factors (*P* < 0.1) were included

^*^The bold values indicate *P-*value < 0.05

All these findings suggest that patients with an initial phenotype of focal inflammation were more likely to experience chronic pouchitis when the focal inflammation subsequently progressed to diffuse inflammation of the pouch body.

### Predictability of initial phenotypes and contributing factors for pouch failure

The rate of pouch failure was 4.9%, and DLI and pouch excision were conducted in 4.9% and 0.8% of cases, respectively (Table 2). A KM curve showed that the 10-year pouch failure-free survival rate was 95.3% (95% CI 92.9–97.8%) (Fig. 2c).

We found that patients with an initial phenotype of inlet involvement had an increased risk of pouch failure over time (*P* < 0.001, *n* = 376) (Fig. 2d, Fig. S6). Similar trends were observed in asymptomatic patients at the initial pouchoscopy (*n* = 121) (Fig. S7). The Cox proportional hazards model including the 4 factors with the smallest *P*-values (Table S8) showed that an initial phenotype of inlet involvement was a significant predictor of pouch failure (HR, 6.3; 95% CI 1.3–30.8;* P* = 0.023) (Table 3).

Our subgroup analysis of patients with an initial phenotype of inlet involvement showed that the rate of inlet ulcers was 2 times higher in patients with pouch failure than in those without it (*P* = 0.055) (Table S9). KM curves showed that patients with inlet ulcers had a significantly increased risk of pouch failure over time (*P* = 0.034) (Fig. S8). Whilst the number of inflammatory phenotypes in the initial endoscopy did not differ, the number as well as the rate of pouch-related fistula in the overall phenotypes was significantly higher in patients with pouch failure than in those without it. Among 9 patients with an initial phenotype of inlet involvement who later developed pouch failure, 5 patients (56%) and 1 patient (11%) developed pouch-related fistula and inlet stenosis, respectively (Table S9). Images of a representative case are shown in Fig. S9.

In terms of contributing factors to pouch failure, the rates of AL involvement, inlet involvement, and pouch-related fistula as overall phenotypes were significantly higher in patients who experienced pouch failure than in those who did not (Table S10). Logistic regression analysis showed that the risk of pouch failure was significantly associated with an overall phenotype of pouch-related fistula (OR, 13.8; 95% CI 3.0–67.9; *P* < 0.001) (Table 4).

This result demonstrated that inlet involvement, especially inlet ulcer, is a predictor for pouch failure and is likely to be complicated by pouch-related fistula. Furthermore, our cohort had a lower risk of pouch excision than that of the Chicago Classification study (10.7%) [6], which may be attributable to the low frequency of pouch-related fistula.

### Contributing factors for acute pouchitis

The frequency of acute pouchitis was 31% (Table 2). A KM curve showed the 10-year acute pouchitis-free survival rate to be 85.5% (95% CI 80.5–90.8%) (Fig. S10a).

Our initial phenotype analysis showed no specific initial phenotype associated with the risk of acute pouchitis (Fig. S10). The frequencies of AL involvement, inlet involvement, and diffuse inflammation of the pouch body as overall phenotypes were significantly higher in patients with acute pouchitis than in those without it (Table S11). Logistic regression analysis showed that the risk of acute pouchitis was significantly associated with hand-sewn anastomosis (OR, 4.2; 95% CI 2.3–8.1; *P* < 0.001) and an overall phenotype of inlet involvement (OR, 4.1; 95% CI 2.0–8.6; *P* < 0.001) (Table S12).

### Correlation between endoscopic phenotype and clinical subscore of PDAI

To evaluate the correlation between clinical symptoms and endoscopic phenotypes, we combined 672 scopes with available clinical subscores of the PDAI regardless of the postoperative timing. Our analysis showed that patients with a subscore of 4–6 had a significantly higher rate of multiple inflammatory phenotypes (82%) than did patients with a subcore of 0 or 1–3 (52% and 52%, respectively). The proportions of patients with AL involvement, inlet involvement, and diffuse inflammation were significantly higher, whereas the rates of normal phenotype and focal inflammation were significantly lower in patients with a subscore of 4–6 than in those with a subscore of 0 or 1–3 (Table 5).

**Table 5:** Clinical subscore of pouchitis disease activity index and endoscopic phenotypes

Symptoms					
Variable	*N*	Clinical subscore (0), *N* = 271^1^	Clinical subscore (1–3), *N* = 339^1^	Clinical subscore (4–6), *N* = 62^1^	*P*-value^2^
Normal	672	70 (26%)	54 (16%)	2 (3.2%)	** < 0.001**
Afferent limb involvement	465	16 (10%)	55 (22%)	22 (43%)	** < 0.001**
Inlet involvement	538	42 (23%)	114 (39%)	45 (75%)	** < 0.001**
Diffuse involvement of the pouch body	669	19 (7.0%)	67 (20%)	34 (55%)	** < 0.001**
Focal involvement of the pouch body	669	172 (63%)	201 (60%)	25 (40%)	**0.004**
Cuffitis	466	128 (55%)	110 (53%)	20 (71%)	0.20
Pouch-related fistula	668	7 (2.6%)	11 (3.3%)	2 (3.2%)	0.84
Number of inflammatory phenotypes	672				** < 0.001**
Normal or single phenotype		129 (48%)	163 (48%)	11 (18%)	
Multiple phenotype		142 (52%)	176 (52%)	51 (82%)	

^1^*n* (%)

^2^Fisher's exact test, the bold values indicate *P-*value < 0.05

## Discussion

This study investigated the endoscopic phenotype based on the Chicago Classification and showed a low frequency of pouch-related fistula in our study as compared with that in the previously reported US cohort [6]. Our study found that focal inflammation of the pouch body was the initial phenotype of chronic pouchitis and that it often progressed to diffuse inflammation. We also found that the initial phenotype of pouch failure was inlet involvement, particularly inlet ulceration. Since pouch-related fistula was an independent risk factor for pouch failure, the lower risk of pouch failure in our cohort may be attributable to the low prevalence of pouch-related fistula.

To date, no endoscopic scoring or classification systems have been developed to predict postoperative UC pouch outcomes. Our study demonstrated that the Chicago Classification is a useful tool for predicting chronic pouchitis and pouch failure. When performing the initial postoperative pouchoscopy, focal pouch inflammation may need careful endoscopic observation to monitor specific phenotype transitions even in asymptomatic patients. The phenotype transition analysis recently published from the University of Chicago showed that 26% of patients with focal inflammation subsequently developed diffuse inflammation [13]. Our data also showed that 30% of patients with an initial phenotype of focal inflammation progressed to diffuse pouch inflammation when chronic pouchitis developed, supporting the notion that this is a high-risk phenotype transition for chronic pouchitis.

Whilst a meta-analysis showed that 6.4% and 5.5% of patients with UC showed pouch fistula and pouch failure, respectively [14], these percentages vary among countries. For example, a retrospective study conducted at the University of Chicago showed that 18.6% of patients with UC had pouch-related fistula and 10.7% required pouch excision [6]. Meanwhile, a previous Japanese multicenter study showed that 2.6% of patients with UC experienced pouch failure and approximately half of the patients had a fistula/abscess [15]. Consistently, we here showed that fewer than 5% of our patients had pouch failure and pouch-related fistula, suggesting that Japanese patients with IPAA may have favorable outcomes.

Compared to the University of Chicago cohort [6], our cohort had a higher rate of hand-sewn anastomosis (21.5% vs. 58%) and almost half of our patients were preoperatively treated with apheresis. While more than 20% of patients in the former group used tumor necrosis factor inhibitors and immunomodulators postoperatively, only up to 6% of patients in the latter group used these medications. The differences in these managements between Japan and the US may be associated with the lower rate of pouch-related fistula in our cohort. Patient selection for surgery is also important for pouch outcomes, and the differential diagnosis between UC and CD may be a crucial step in ensuring optimal patient selection. A systematic review revealed that multiple guidelines have been published by international IBD societies, with notable variations in recommendations between them [16]. Both the Japanese guideline [17] and the US guideline [18] emphasize the importance of colonoscopy and pathologic evaluation in the diagnosis of UC. For CD, while both the Japanese and US guidelines [19] describe discontinuous involvement with skip lesions in the gastrointestinal tract, the Japanese guideline focuses on the endoscopic and radiographic findings of CD [17], encouraging further imaging studies that improve the accuracy of differential diagnosis between UC and CD. Indeed, data from the Cleveland Clinic Foundation on patients with UC (85%) and indeterminate colitis (15%) undergoing IPAA showed that the rate of diagnostic revision to CD was 7% (184/2,814) [20]. In contrast, a previous Japanese multicenter study of UC reported a lower rate of postoperative CD diagnosis of only 0.7% (16/2376) [15]. Therefore, we believe that the lower rate of pouch-related fistula in Japan may reflect the higher accuracy of preoperative differential diagnosis of IBD based on the Japanese IBD guideline [17]. Since CDLPI [11, 21] is associated with a unique dysbiosis [12] and colonic goblet cell metaplasia [7], these pathologic differences between patients in Japan and other countries are also implicated. Whilst no consistent predictors of late fistula development have been reported [22], our study identified inlet involvement, specifically ulceration, as a predictor of pouch failure. Although this is an important finding that may improve endoscopic pouch monitoring, further studies with larger sample sizes are warranted to confirm our findings.

Our analysis of secondary outcomes showed a limitation in predicting acute pouchitis using the Chicago Classification. We also found that hand-sewn anastomosis was significantly associated with acute pouchitis. Conversely, hand-sewn anastomosis was inversely associated with the risk of chronic pouchitis, and an overall phenotype of cuffitis was a significant contributing factor to chronic pouchitis. A previous study reported that the rate of phenotype transition from cuffitis to diffuse inflammation of the pouch body was 24.8% [13]. In addition, a retrospective analysis assessing clinical symptoms and endoscopic phenotypes of the pouch showed that patients with cuffitis had the highest symptom subscore of the PDAI [23]. These findings suggest that the residual rectal cuff resulting from stapled anastomosis may contribute to chronic inflammatory pouch conditions as well as chronic symptoms in UC patients with IPAA. Thus, the impact of endoscopic phenotypes on clinical symptoms of pouchitis was also investigated. Consistent with previous findings [23], our analysis regarding the clinical subscore of the PDAI showed that patients with a lower subscore were more likely to have a normal phenotype or focal inflammation, whereas patients with a higher subscore often had multiple inflammatory phenotypes, suggesting that the overlap of inflammatory phenotypes may deteriorate their clinical symptoms and normalization of the pouch would be associated with improvement in patients’ QOL.

This study has several strengths and limitations. As a major strength, to the best of our knowledge, this is the first multicenter study that included approximately 400 UC patients with IPAA from 12 IBD centers in Japan and demonstrated the prevalence of each endoscopic phenotype based on the Chicago Classification and their predictability for chronic pouchitis and pouch failure. However, we must acknowledge the limitations of the study due to its retrospective nature. For instance, chronic pouchitis was not necessarily classified into antibiotic-dependent and antibiotic-refractory chronic pouchitis. Endoscopic findings at each anatomic location of the pouch (eg, the afferent limb or rectal cuff) were not always available, and this issue may have affected the prevalence of AL involvement or cuffitis. In addition, routine endoscopic evaluations at 3 and 10 years after ileostomy takedown were not always evaluated because pouch surveillance protocols vary among hospitals. Details regarding endoscopic monitoring methods have not been fully evaluated as our data set does not include the indication for pouchoscopy. In this study, collaborating investigators at each participating hospital reviewed the endoscopic images and reports from their respective institutions. However, we acknowledge that central reading or consensus review by more than one endoscopist would be the ideal approach for evaluating pouchoscopy findings. Finally, although we have shown the predictability of the Chicago Classification for pouch outcomes, changes in postoperative treatments for inflammatory pouch conditions may affect these outcomes. Therefore, a prospective study with a standard operating protocol that is commonly established at each participating institution is ideal to minimize such biases.

In conclusion, our analysis using the Chicago Classification showed a lower frequency of pouch-related fistula, which may have led to a favorable pouch outcome in our cohort. We also found that this classification is predictive of chronic pouchitis and pouch failure in patients with UC. If focal inflammation of the pouch body at the initial postoperative pouchoscopy subsequently progresses to diffuse inflammation or develops other inflammatory phenotypes, careful monitoring may be required for early detection of chronic pouchitis. The initial phenotype of inlet involvement, especially ulceration, may also be a warning sign of pouch failure. Future investigations are needed to better understand how the Chicago Classification can be appropriately applied to the postoperative management of UC patients with IPAA.

## Supplementary Information

Below is the link to the electronic supplementary material.

Supplementary file1 (DOCX 22 KB)

Supplementary file2 (PDF 1625 KB)

Supplementary file3 (DOCX 109 KB)
