# Supplementary material for: Prevalence and predictability of the Chicago Classification of Pouchitis in ulcerative colitis: a multicenter study in Japan
Source: J Gastroenterol. 2025 Mar 6;60(6):715–26. doi: 10.1007/s00535-025-02231-1 (PMC12095421; doi:10.1007/s00535-025-02231-1)
Supplement: Supplementary file 1 — Supplementary file1 (DOCX 22 KB) [file 535_2025_2231_MOESM1_ESM.docx]

**Supplementary Figure Legends**

**Fig. S1.** Flowchart of the eligibility assessment of patients with ulcerative colitis for this study.

**Fig. S2.** Kaplan-Meier curves evaluating chronic pouchitis-free survival (n = 317) for **(a)** overall population and **(b)** normal phenotype, **(c)** afferent limb involvement, **(d)** inlet involvement, **(e)** diffuse inflammation, **(f)** focal inflammation of the pouch body, **(g)** cuffitis, **(h)** pouch-related fistula, and **(i)** number of inflammatory phenotypes observed at the initial postoperative pouchoscopy.

**Fig. S3.** Kaplan-Meier curves evaluating chronic pouchitis-free survival in asymptomatic patients (n = 119) for **(a)** overall population and **(b)** normal phenotype, **(c)** afferent limb involvement, **(d)** inlet involvement, **(e)** diffuse inflammation, **(f)** focal inflammation of the pouch body, **(g)** cuffitis, **(h)** pouch-related fistula, and **(i)** number of inflammatory phenotypes observed at the initial postoperative pouchoscopy.

**Fig. S4.** Subgroup analysis of patients with focal inflammation of the pouch body at the initial postoperative pouchoscopy. Kaplan-Meier curves comparing chronic pouchitis-free survival between patients who had erythema/edema at the distal pouch body (DP) and those who did not.

**Fig. S5.** Representative endoscopic images of the progression from focal inflammation to diffuse inflammation of the pouch body. **(a-b)** Ulcerations with erythema in the distal pouch were noted on initial postoperative pouchoscopy. **(c-d)** Diffuse inflammation of the pouch body was observed 6 years later. This patient was diagnosed with chronic pouchitis. **(e-f)** The diffuse inflammation of the pouch body was improved with metronidazole and oral aminosalicylates.

**Fig. S6.** Kaplan-Meier curves evaluating pouch failure-free survival (n = 376) for **(a)** overall population and **(b)** normal phenotype, **(c)** afferent limb involvement, **(d)** inlet involvement, **(e)** diffuse inflammation, **(f)** focal inflammation of the pouch body, **(g)** cuffitis, **(h)** pouch-related fistula, and **(i)** number of inflammatory phenotypes observed at the initial postoperative pouchoscopy.

**Fig. S7.** Kaplan-Meier curves evaluating pouch failure-free survival in asymptomatic patients (n = 121) for **(a)** overall population and **(b)** normal phenotype, **(c)** afferent limb involvement, **(d)** inlet involvement, **(e)** diffuse inflammation, **(f)** focal inflammation of the pouch body, **(g)** cuffitis, **(h)** pouch-related fistula, and **(i)** number of inflammatory phenotypes observed at the initial postoperative pouchoscopy.

**Fig. S8.** Subgroup analysis of patients with inlet involvement at the initial postoperative pouchoscopy. Kaplan-Meier curves comparing pouch-failure-free survival between patients who had inlet (IL) ulceration and those who did not.

**Fig. S9.**

Representative endoscopic images of an initial phenotype of inlet involvement. **(a-b)** Erosions and erythema in the inlet and cuff were noted at the initial postoperative pouchoscopy. **(c-d)** A pouch-related fistula developed from the pouch 1 year later. **(e-f)** A seton was placed and diverting loop ileostomy was performed for the pouch-related fistula.

**Fig. S10.** Kaplan-Meier curves evaluating acute pouchitis-free survival (n = 291) for **(a)** the overall population and those with **(b)** a normal phenotype, **(c)** afferent limb involvement, **(d)** inlet involvement, **(e)** diffuse inflammation, **(f)** focal inflammation of the pouch body, **(g)** cuffitis, **(h)** pouch-related fistula, and **(i)** number of inflammatory phenotypes observed at the initial postoperative pouchoscopy.
