# Supplementary material for: Prevalence and predictability of the Chicago Classification of Pouchitis in ulcerative colitis: a multicenter study in Japan
Source: J Gastroenterol. 2025 Mar 6;60(6):715–26. doi: 10.1007/s00535-025-02231-1 (PMC12095421; doi:10.1007/s00535-025-02231-1)
Supplement: Supplementary file 2 — Supplementary file2 (PDF 1625 KB) [file 535_2025_2231_MOESM2_ESM.pdf]

Fig. S1

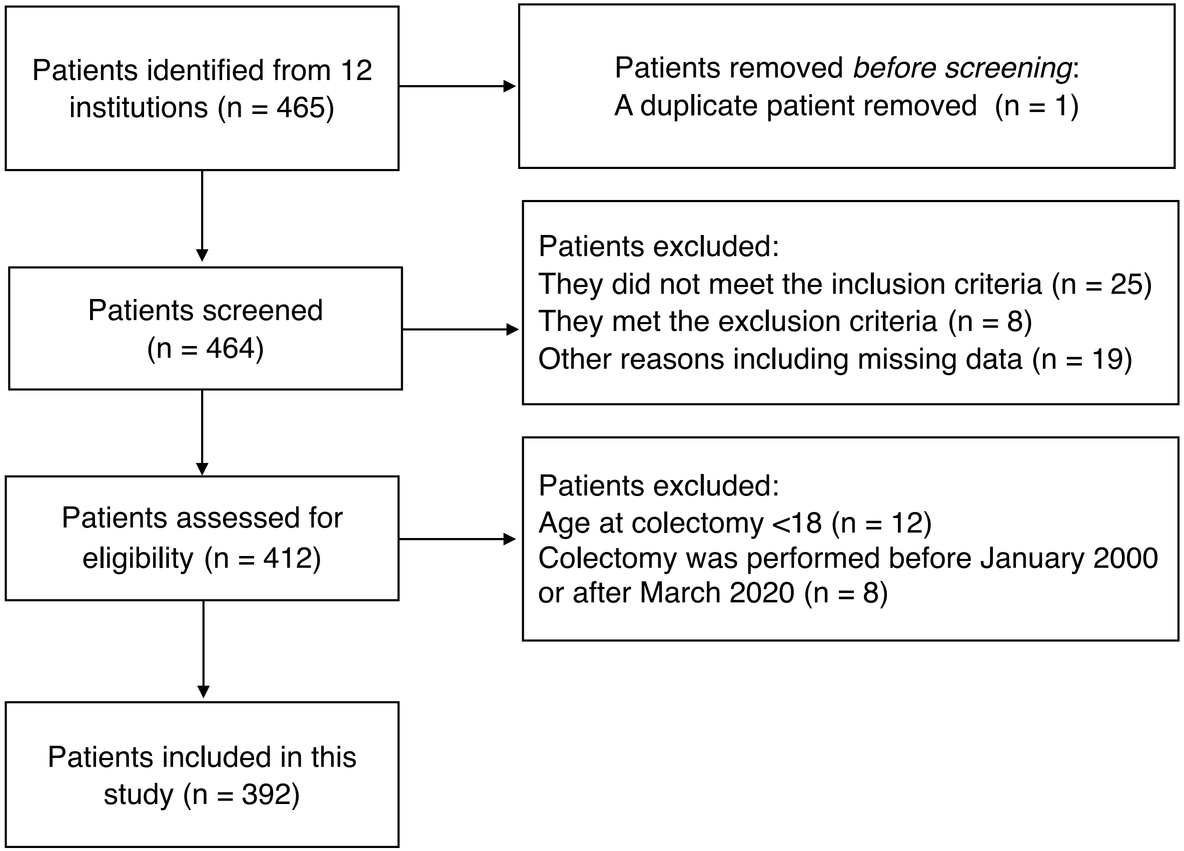
Patients identified from 12 institutions (n = 465)
Patients removed *before screening*: A duplicate patient removed (n = 1)
Patients screened (n = 464)
Patients excluded:
They did not meet the inclusion criteria (n = 25)
They met the exclusion criteria (n = 8)
Other reasons including missing data (n = 19)
Patients assessed for eligibility (n = 412)
Patients excluded:
Age at colectomy <18 (n = 12)
Colectomy was performed before January 2000 or after March 2020 (n = 8)
Patients included in this study (n = 392)

Fig. S2 All patients included in the analysis (n = 317)

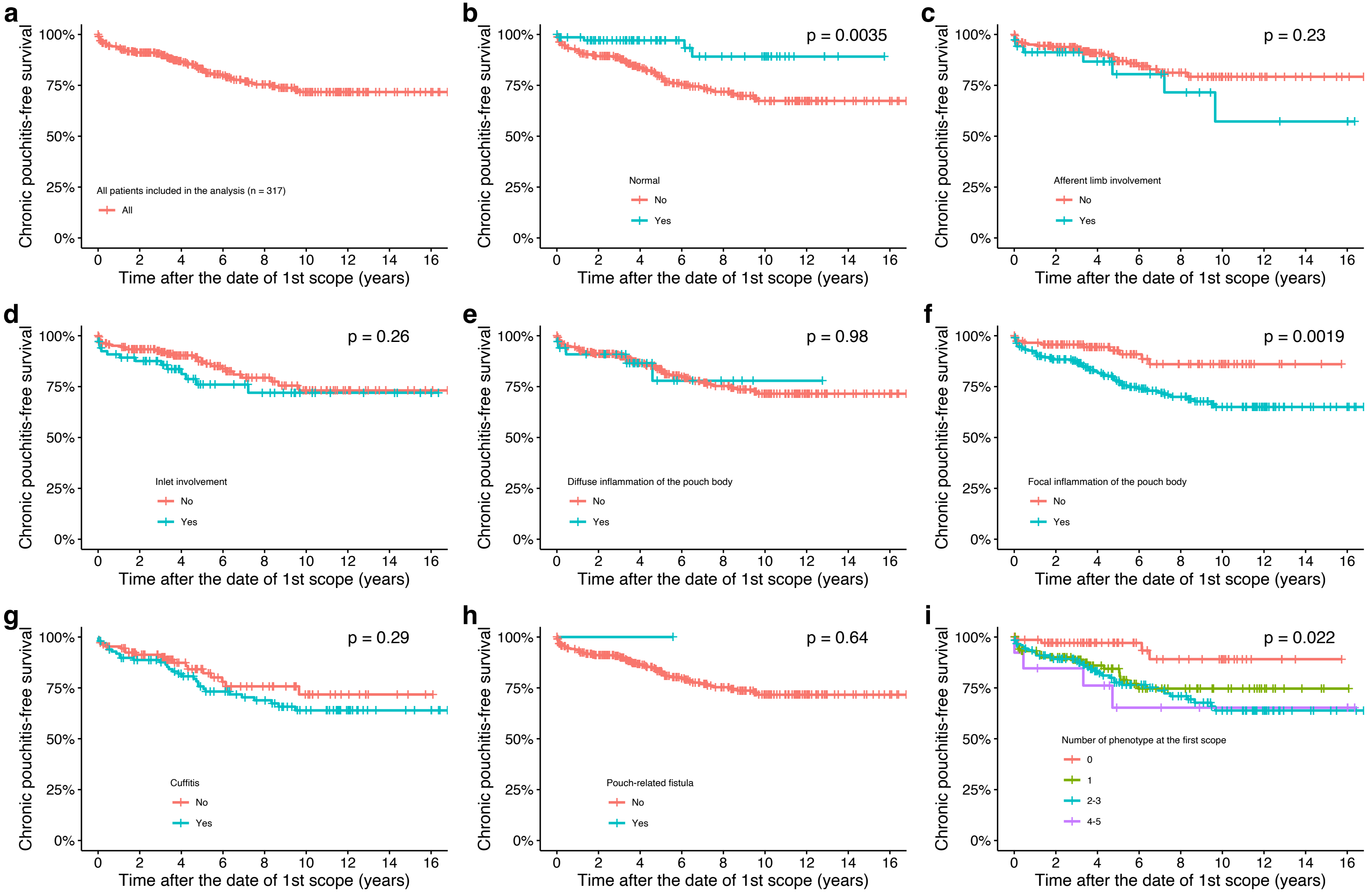

Fig. S3 Asymptomatic patients at the initial pouchoscopy (n = 119)

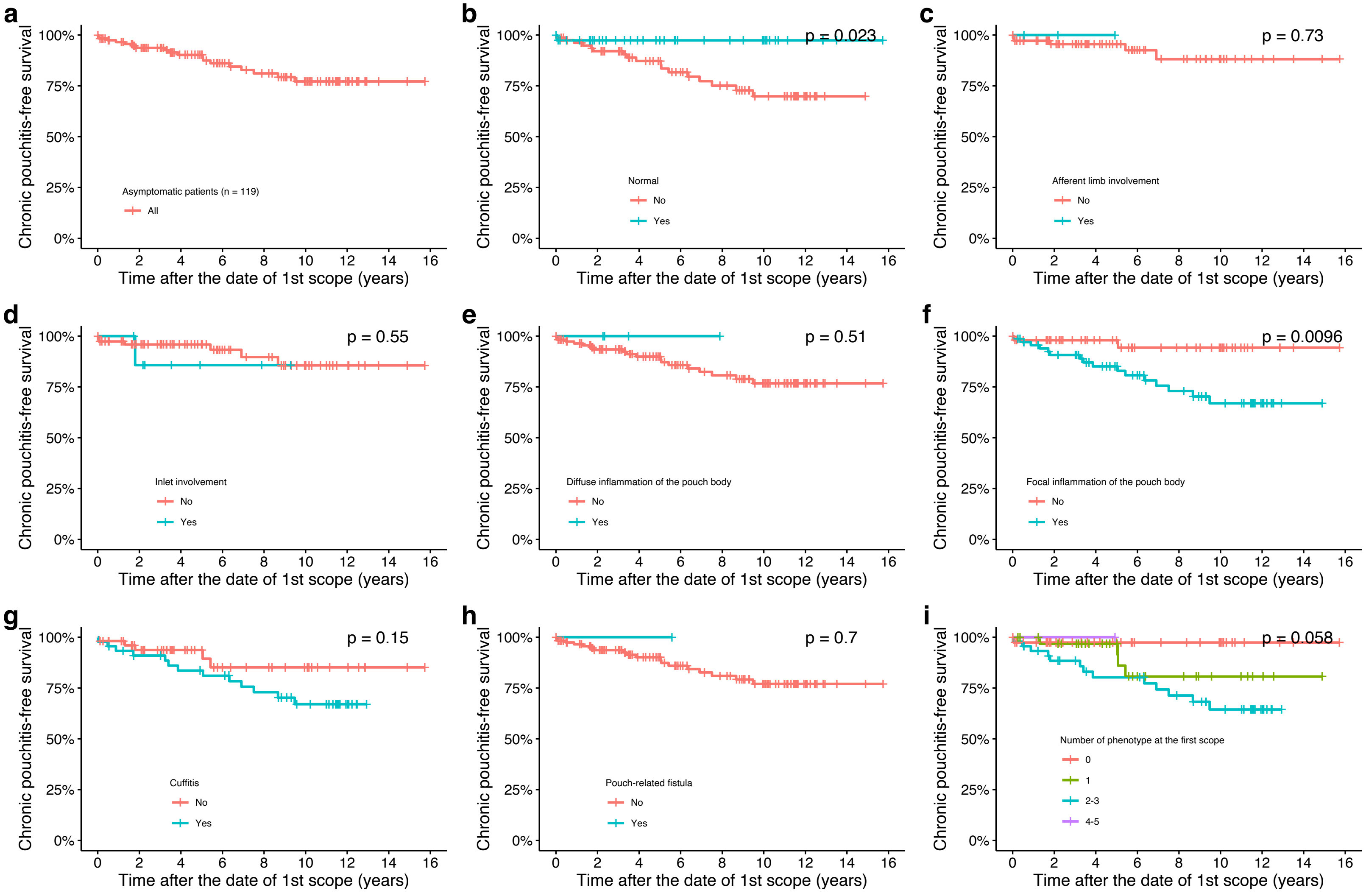

Fig. S4

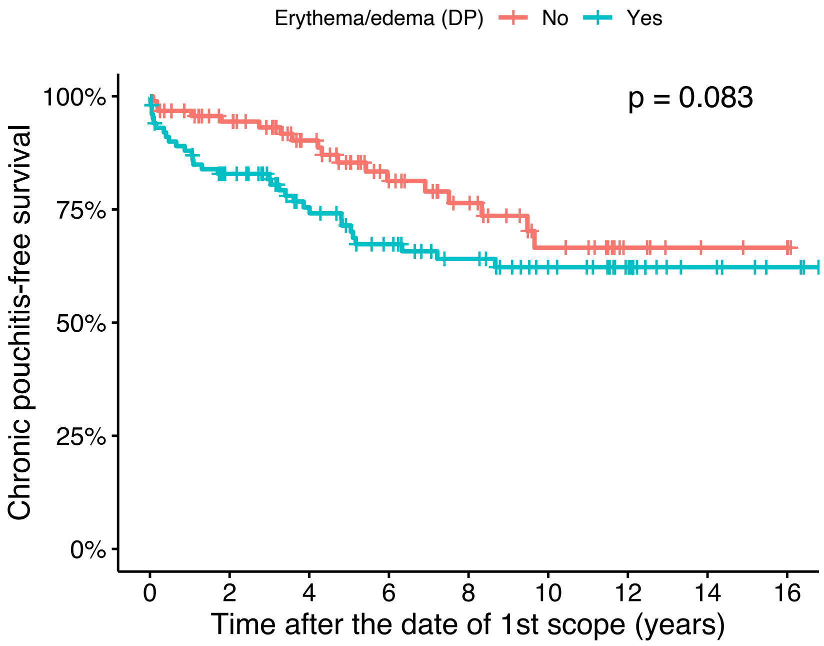

Erythema/edema (DP)
No
Yes
p = 0.083
100%
75%
50%
25%
0%
Chronic pouchitis-free survival
0
2
4
6
8
10
12
14
16
Time after the date of 1st scope (years)

Fig. S5

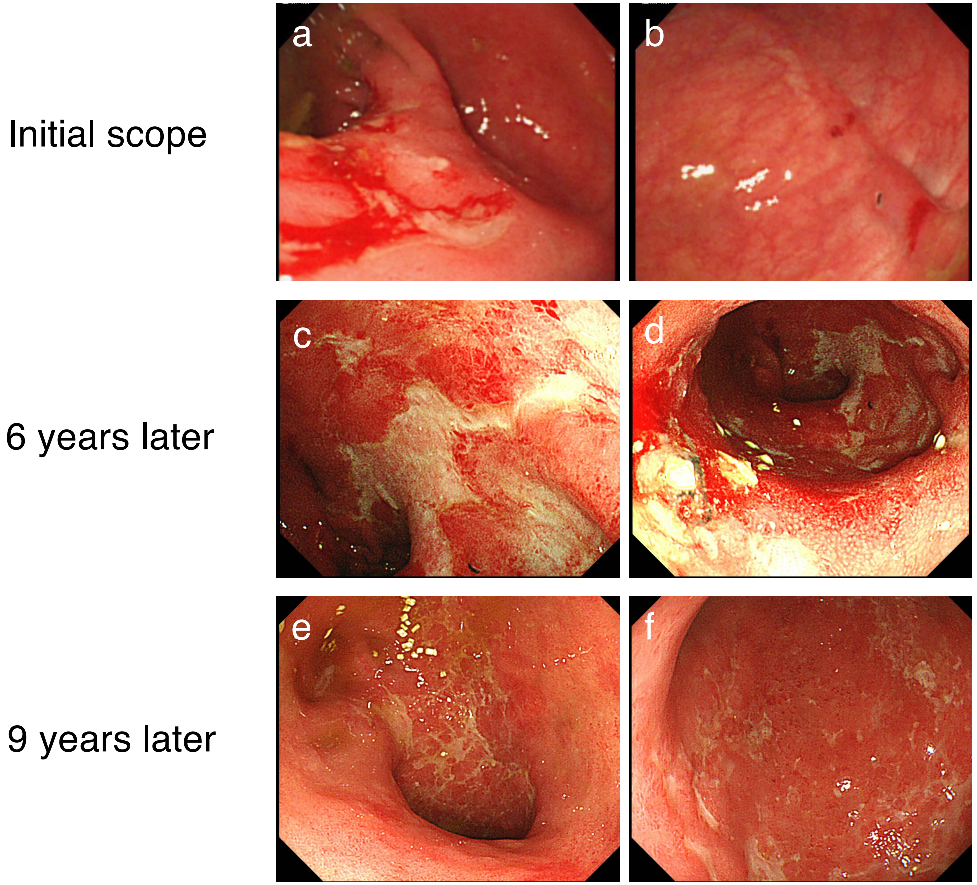

Fig. S6 All patients included in the analysis (n = 376)

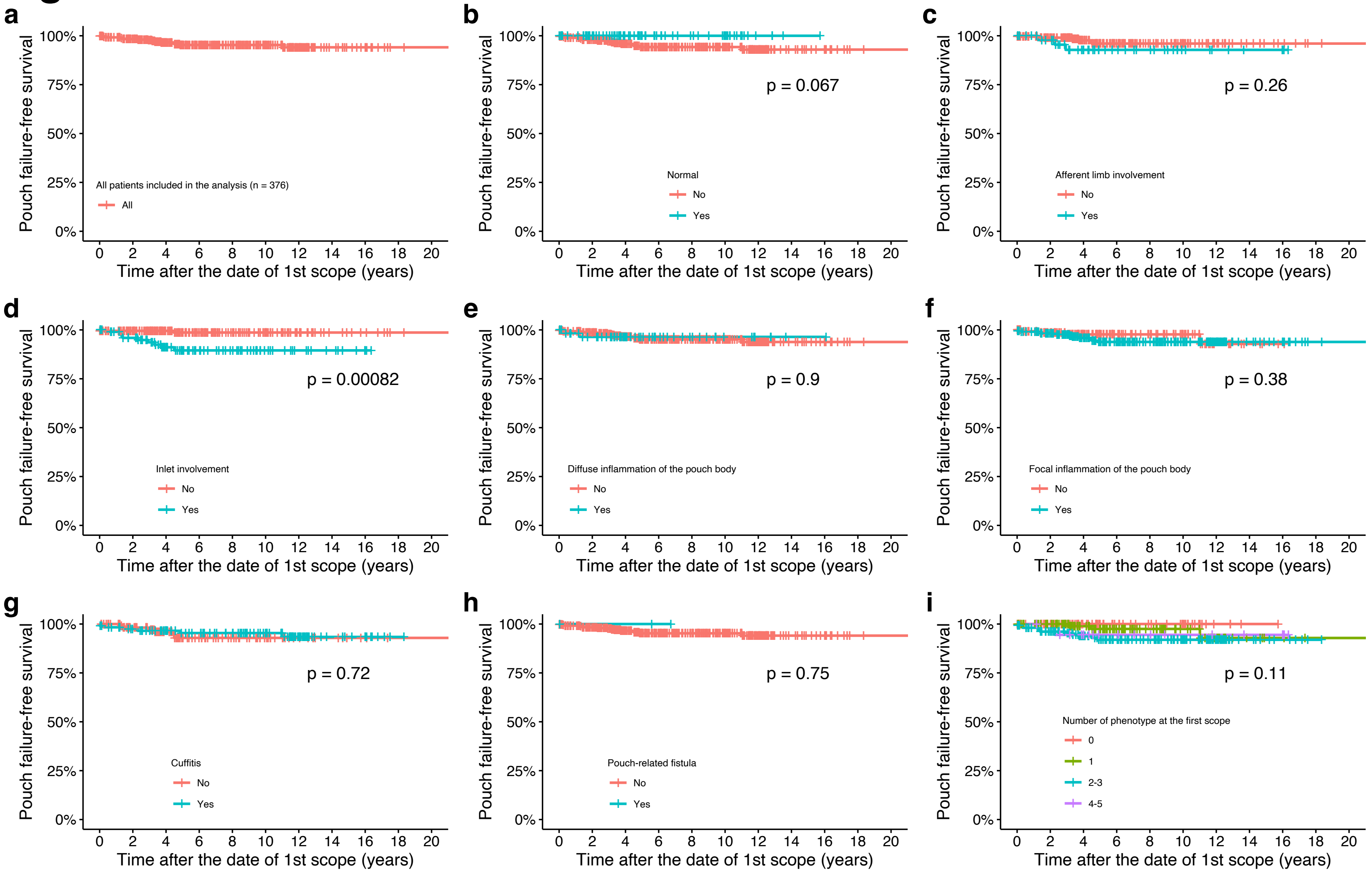

Fig. S7 Asymptomatic patients at the initial pouchoscopy (n = 121)

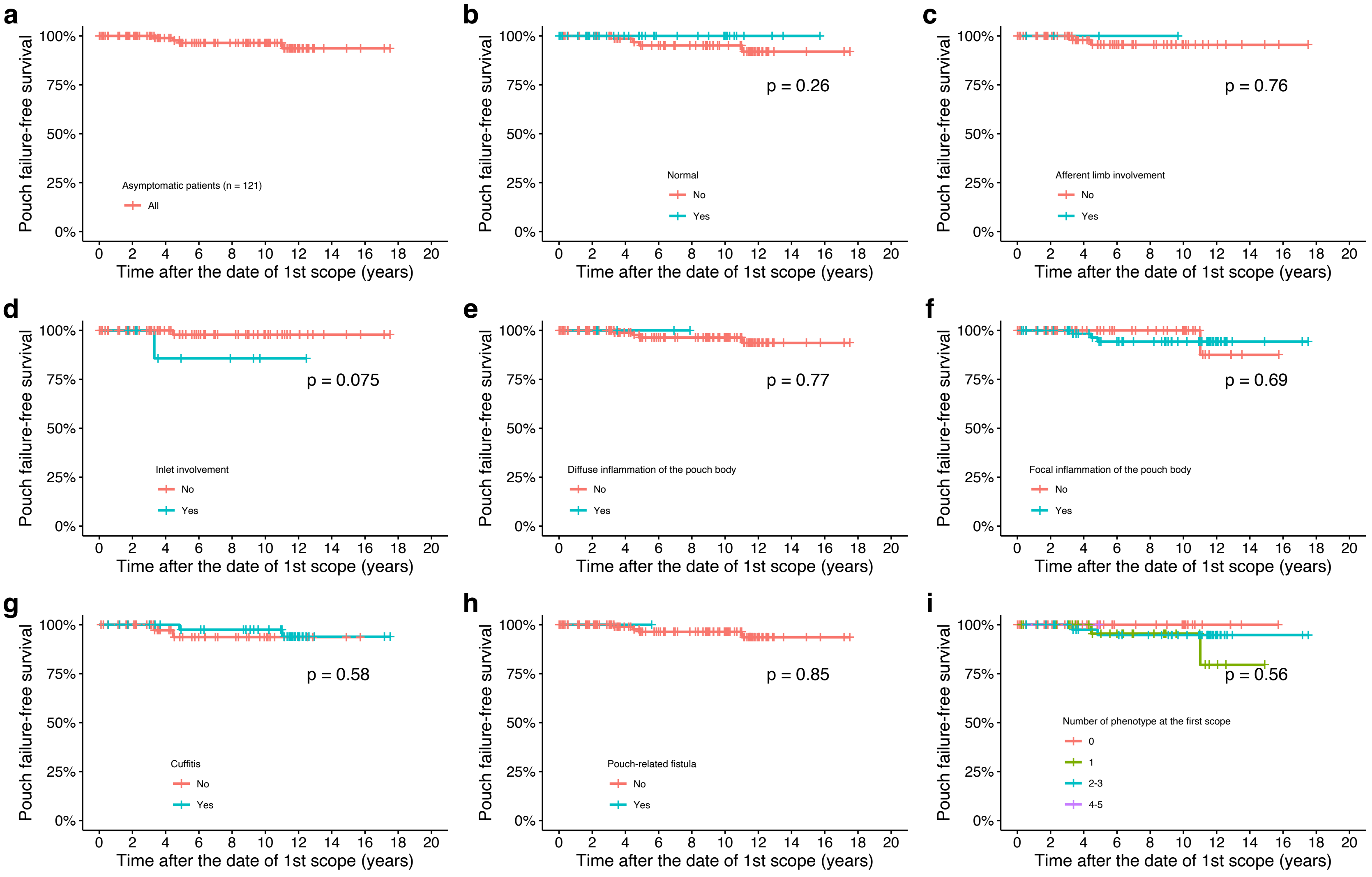

Fig. S8

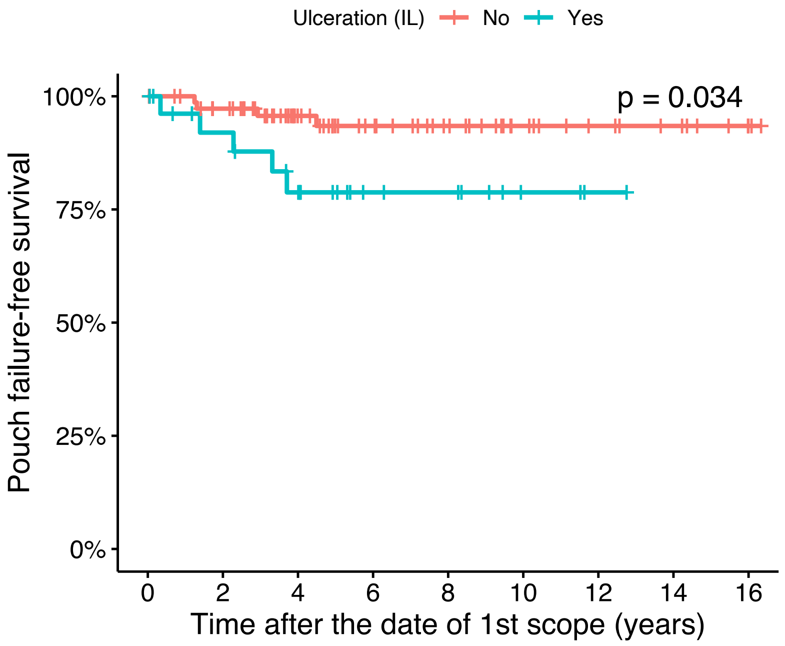
Ulceration (IL)
No
Yes
p = 0.034
100%
75%
50%
25%
0%
Pouch failure-free survival
0
2
4
6
8
10
12
14
16
Time after the date of 1st scope (years)

Fig. S9

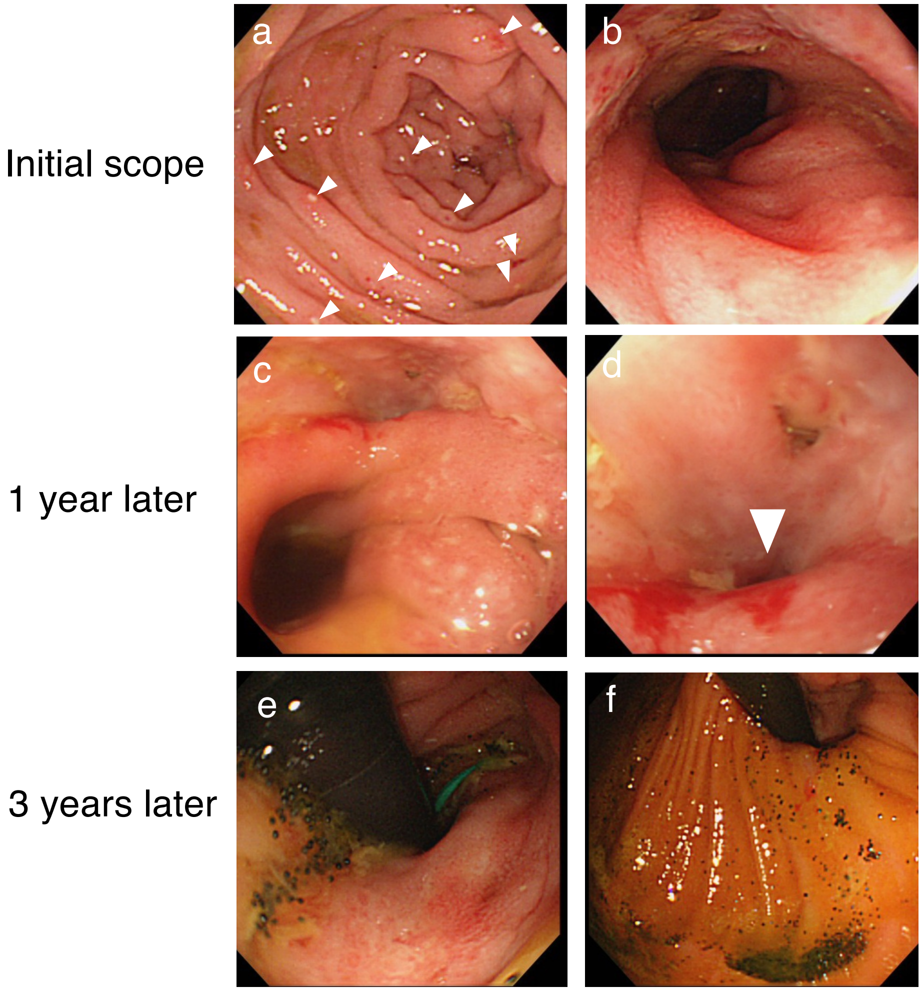

Fig. S10

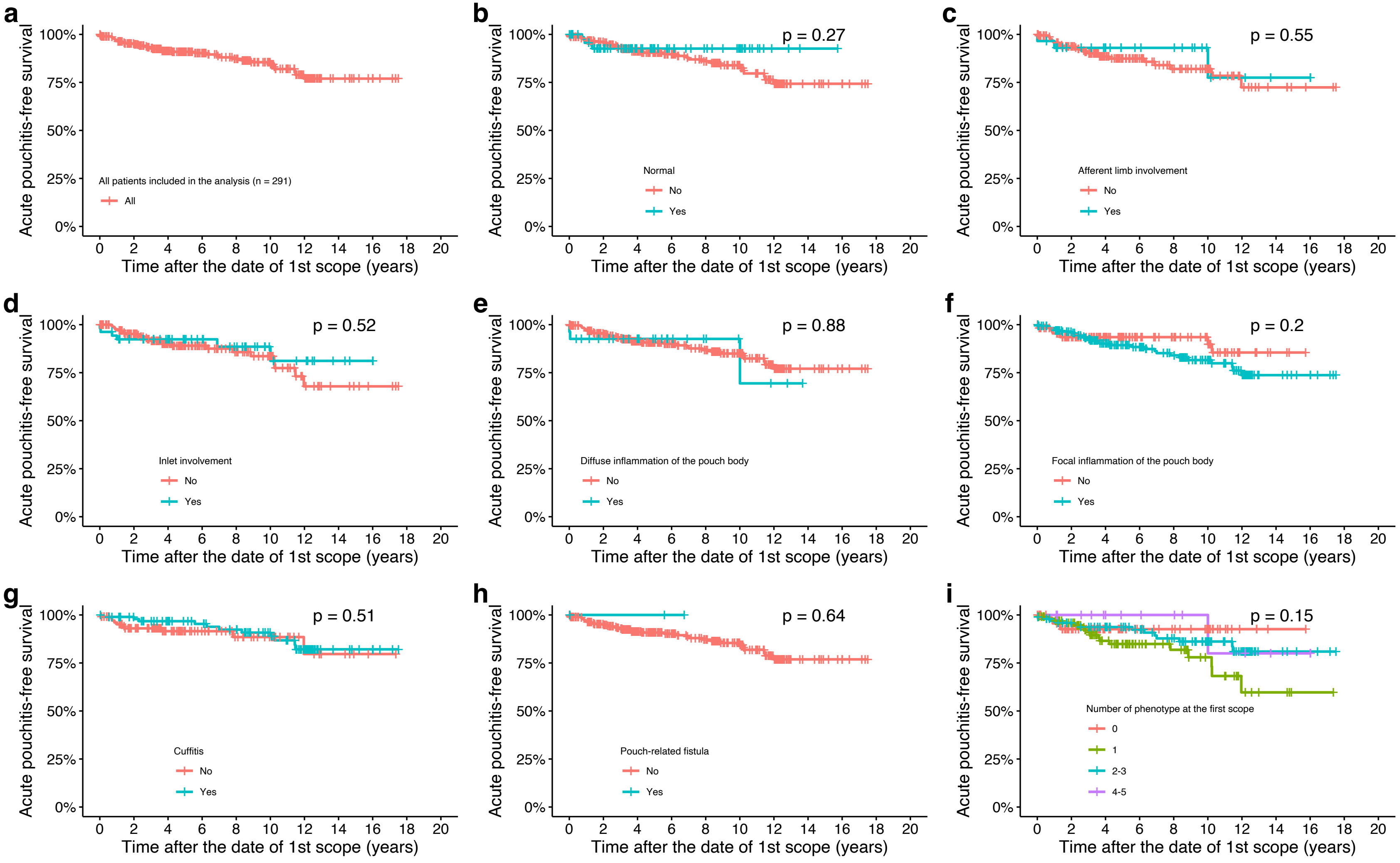
a
Acute pouchitis-free survival
100%
75%
50%
25%
0%
All patients included in the analysis (n = 291)
All
0 2 4 6 8 10 12 14 16 18 20
Time after the date of 1st scope (years)
b
p = 0.27
Normal
No
Yes
c
p = 0.55
Afferent limb involvement
No
Yes
d
p = 0.52
Inlet involvement
No
Yes
e
p = 0.88
Diffuse inflammation of the pouch body
No
Yes
f
p = 0.2
Focal inflammation of the pouch body
No
Yes
g
p = 0.51
Cuffitis
No
Yes
h
p = 0.64
Pouch-related fistula
No
Yes
i
p = 0.15
Number of phenotype at the first scope
0
1
2-3
4-5
