# Supplementary material for: Prevalence and predictability of the Chicago Classification of Pouchitis in ulcerative colitis: a multicenter study in Japan
Source: J Gastroenterol. 2025 Mar 6;60(6):715–26. doi: 10.1007/s00535-025-02231-1 (PMC12095421; doi:10.1007/s00535-025-02231-1)
Supplement: Supplementary file 3 — Supplementary file3 (DOCX 109 KB) [file 535_2025_2231_MOESM3_ESM.docx]

**Table S1. Affiliations and Number of Cases at the 12 Participating Hospitals**

| **No.** | **Institutions** | **Prefecture** | **Department** | **Number of registered cases** | **Number of included cases** |
| --- | --- | --- | --- | --- | --- |
| 1 | University of Tsukuba | Ibaraki | Department of Gastroenterology | 51 | 47 |
| 2 | Hiroshima University Hospital | Hiroshima | Department of Gastroenterology and Metabolism | 80 | 74 |
| 3 | Hyogo Medical University | Hyogo | Department of Gastroenterological Surgery | 76 | 70 |
| 4 | Institute of Science Tokyo | Tokyo | Department of Gastroenterology and Hepatology | 75 | 44 |
| 5 | Tohoku University | Sendai | Department of Surgery | 71 | 51 |
| 6 | Yokohama Municipal Citizen's Hospital | Yokohama | Department of Inflammatory Bowel Disease | 40 | 40 |
| 7 | Asahikawa Medical University | Asahikawa | Department of Medicine | 24 | 20 |
| 8 | Keio University | Tokyo | Center for Preventive Medicine | 17 | 17 |
| 9 | Nara Medical University | Kashihara | Department of Surgery | 17 | 16 |
| 10 | Saga University | Saga | Department of Internal Medicine | 11 | 11 |
| 11 | Tsujinaka Hospital Kashiwanoha | Chiba | Department of Gastroenterology | 2 | 2 |
| 12 | Kansai Medical University | Osaka | The Third Department of Internal Medicine | 1 | 0 |

Table S2. Fistula type and treatments

| **Characteristic** | **N = 19**^1^ |
| --- | --- |
| **Fistula type**  Perianal fistula | 11 (58%) |
| Rectovaginal/anovaginal fistula | 2 (11%) |
| Enterocutaneous fistula | 2 (11%) |
| Enterovesical fistula | 0 (0%) |
| Enteroenteric fistula | 0 (0%) |
| Fistula from the pouch | 6 (32%) |
| Unknown (Fistula type) | 1 (5.3%) |
| Others (Fistula type) | 1 (5.3%) |
| **Treatments** |  |
| Antibiotics | 14 (74%) |
| Biologics | 0 (0%) |
| Incision and drainage | 5 (26%) |
| Seton drainage | 9 (47%) |
| Diverting loop ileostomy | 6 (32%) |
| Pouch excision | 1 (5.3%) |
| Fistulotomy/fistulectomy | 2 (11%) |
| Unknown (Fistula treatment) | 0 (0%) |
| Others (Fistula treatment) | 6 (32%) |
| ^1^n (%) | |

Table S3. Postoperative Management for Crohn’s Disease-Like Pouch Inflammation (CDLPI)

|  | | **CDLPI** | |  |
| --- | --- | --- | --- | --- |
| **Variable** | **N** | **No**,  N = 282^1^ | **Yes**,  N = 110^1^ | **P-value**^2^ |
| **Loperamide** | 390 | 199 (71%) | 83 (75%) | 0.45 |
| **Metronidazole/ciprofloxacin** | 390 | 143 (51%) | 76 (69%) | 0.001 |
| **Oral aminosalicylates** | 390 | 27 (9.6%) | 17 (15%) | 0.11 |
| **Topical aminosalicylates** | 390 | 28 (10%) | 8 (7.3%) | 0.44 |
| **Oral steroids** | 390 | 22 (7.9%) | 10 (9.1%) | 0.68 |
| **Topical steroids** | 390 | 37 (13%) | 13 (12%) | 0.87 |
| **Tumor necrosis factor inhibitors** | 390 | 12 (4.3%) | 10 (9.1%) | 0.086 |
| **Immunomodulators** | 390 | 4 (1.4%) | 4 (3.6%) | 0.23 |
| **Ustekinumab** | 390 | 2 (0.7%) | 1 (0.9%) | >0.99 |
| **Vedolizumab** | 390 | 5 (1.8%) | 3 (2.7%) | 0.69 |
| **Janus kinase inhibitors** | 390 | 1 (0.4%) | 2 (1.8%) | 0.19 |
| **Calcineurin inhibitors** | 390 | 2 (0.7%) | 0 (0%) | >0.99 |
| **No postoperative treatments** | 390 | 21 (7.5%) | 2 (1.8%) | 0.032 |
| **Diverting loop ileostomy** | 389 |  |  | <0.001 |
| 1. No |  | 273 (98%) | 97 (88%) |  |
| 2. Yes |  | 6 (2.2%) | 13 (12%) |  |
| **Pouch excision** | 387 |  |  | 0.021 |
| 1. No |  | 279 (100%) | 105 (97%) |  |
| 2. Yes |  | 0 (0%) | 3 (2.8%) |  |
| ^1^Median (IQR) or Frequency (%) | | | | |
| ^2^Fisher's exact test | | | | |

Table S4. Comparison of Patient Characteristics Between Our Cohort and the External Cohort (University of Chicago)

| **Variables** | **Multicenter Japan** | **University of Chicago** | **p-value** |
| --- | --- | --- | --- |
| **Age at diagnosis (≥18 y/o)** | 340/389 (87.4%) | 290/376 (77.1%) | <0.001 |
| **Disease duration until surgery (≥7 yrs)** | 166/389 (42.7%) | 133/375 (35.5%) | 0.049 |
| **Body mass index (≥25)** | 47/391 (12%) | 201/365 (55.1%) | <0.001 |
| **Gender (Male)** | 224/392 (57.1%) | 217/382 (56.8%) | 0.982 |
| **Disease extent (Extensive colitis)** | 307/379 (81%) | 269/316 (85.1%) | 0.182 |
| **Primary sclerosing cholangitis** | 8/390 (2.1%) | 16/382 (4.2%) | 0.133 |
| **Current smoker** | 11/286 (3.8%) | 11/379 (2.9%) | 0.649 |
| **Stage of ileal pouch-anal-anastomosis (3-stage)** | 99/386 (25.6%) | 190/342 (55.6%) | <0.001 |
| **Anastomosis type (Hand-sewn)** | 219/376 (58.2%) | 82/297 (27.6%) | <0.001 |
| **Preoperative treatments** |  |  |  |
| Tumor necrosis factor inhibitors | 116/373 (31.1%) | 164/353 (46.5%) | <0.001 |
| Immunomodulators | 166/373 (44.5%) | 205/353 (58.1%) | <0.001 |
| Systemic steroids | 335/373 (89.8%) | 324/353 (91.8%) | 0.430 |
| **Indication for surgery** |  |  |  |
| Medically refractory | 266/389 (68.4%) | 303/350 (86.6%) | <0.001 |
| Dysplasia/Colorectal cancer | 85/389 (21.9%) | 44/350 (12.6%) | 0.001 |
| Fulminant colitis | 6/389 (1.5%) | 46/350 (13.1%) | <0.001 |
| Toxic megacolon | 13/389 (3.3%) | 8/350 (2.3%) | 0.521 |
| **Postoperative complications** |  |  |  |
| No postoperative complications | 214/376 (56.9%) | 138/325 (42.5%) | <0.001 |
| Anastomosis leak | 22/376 (5.9%) | 18/325 (5.5%) | 0.988 |
| Pelvic sepsis | 6/376 (1.6%) | 5/325 (1.5%) | 1.000 |
| Abdominal abscess requiring drainage | 19/376 (5.1%) | 42/325 (12.9%) | <0.001 |
| Fistulas or sinus tracts developed until ileostomy takedown | 7/376 (1.9%) | 14/325 (4.3%) | 0.094 |
| **Postoperative treatments** |  |  |  |
| Metronidazole/ciprofloxacin | 219/390 (56.2%) | 311/375 (82.9%) | <0.001 |
| Systemic steroids | 32/390 (8.2%) | 102/375 (27.2%) | <0.001 |
| Tumor necrosis factor inhibitors | 22/390 (5.6%) | 107/375 (28.5%) | <0.001 |
| Immunomodulators | 8/390 (2.1%) | 82/375 (21.9%) | <0.001 |
| **Chicago Classification** |  |  |  |
| Normal | 57/392 (14.5%) | 20/382 (5.2%) | <0.001 |
| Afferent limb involvement | 88/392 (22.4%) | 116/382 (30.4%) | 0.016 |
| Inlet involvement | 168/392 (42.9%) | 157/382 (41.1%) | 0.673 |
| Diffuse inflammation of the pouch body | 106/392 (27%) | 106/382 (27.7%) | 0.889 |
| Focal inflammation of the pouch body | 216/392 (55.1%) | 198/382 (51.8%) | 0.401 |
| Cuffitis | 155/392 (39.5%) | 171/382 (44.8%) | 0.162 |
| Pouch-related fistula | 19/392 (4.8%) | 71/382 (18.6%) | <0.001 |
| **Pouch excision** | 3/387 (0.8%) | 41/382 (10.7%) | <0.001 |

Table S5. Univariate Analysis to Assess Factors Predicting Chronic Pouchitis

(Cox Proportional Hazards Model)

| **Characteristic** | **N** | **HR**^1^ | **95% CI**^1^ | **p-value** |
| --- | --- | --- | --- | --- |
| **Age at diagnosis (yrs)** | 316 | 0.97 | 0.95, 0.99 | 0.007 |
| **Age at colectomy (yrs)** | 317 | 0.96 | 0.94, 0.98 | <0.001 |
| **Disease duration until surgery (yrs)** | 316 | 0.97 | 0.94, 1.00 | 0.079 |
| **Body mass index** | 317 | 0.98 | 0.92, 1.06 | 0.65 |
| **Gender** | 317 |  |  |  |
| Female |  | — | — |  |
| Male |  | 1.06 | 0.64, 1.77 | 0.81 |
| **Montreal classification** | 312 |  |  |  |
| Proctitis |  | — | — |  |
| Left-sided colitis |  | 9,542,328 | 0.00, Inf | >0.99 |
| Extensive colitis |  | 8,987,887 | 0.00, Inf | >0.99 |
| **Primary sclerosing cholangitis** | 316 | 0.73 | 0.10, 5.24 | 0.75 |
| **Current smoker** | 231 | 0.00 | 0.00, Inf | >0.99 |
| **Stage of ileal pouch-anal anastomosis** | 315 |  |  |  |
| 1-stage |  | — | — |  |
| 2-stages |  | 0.51 | 0.29, 0.91 | 0.022 |
| 3-stage |  | 0.32 | 0.14, 0.71 | 0.005 |
| **Anastomosis type** | 304 |  |  |  |
| Staple |  | — | — |  |
| Hand-sewn |  | 0.50 | 0.29, 0.84 | 0.009 |
| **Preoperative treatments** |  |  |  |  |
| Tumor necrosis factor inhibitors | 308 | 1.48 | 0.85, 2.59 | 0.17 |
| Azathioprine/6-mercaptopurine | 308 | 1.57 | 0.94, 2.63 | 0.082 |
| Vedolizumab | 308 | 0.00 | 0.00, Inf | >0.99 |
| Janus kinase inhibitors | 308 | 1.37 | 0.19, 10.0 | 0.76 |
| Oral aminosalicylates | 308 | 1.52 | 0.65, 3.53 | 0.33 |
| Calcineurin inhibitors | 308 | 0.83 | 0.46, 1.51 | 0.54 |
| Systemic steroids | 308 | 2.96 | 0.72, 12.1 | 0.13 |
| Apheresis | 308 | 0.89 | 0.53, 1.49 | 0.66 |
| No preoperative treatments | 308 | 1.47 | 0.20, 10.7 | 0.70 |
| **Indications for colectomy** |  |  |  |  |
| Medically refractory | 315 | 1.70 | 0.88, 3.28 | 0.11 |
| Dysplasia/Colorectal Cancer | 315 | 0.49 | 0.21, 1.14 | 0.098 |
| Fulminant colitis | 315 | 1.03 | 0.14, 7.47 | 0.97 |
| Toxic megacolon | 315 | 1.74 | 0.54, 5.57 | 0.35 |
| Massive hemorrhage | 315 | 0.92 | 0.22, 3.76 | 0.91 |
| Perforation | 315 | 0.90 | 0.22, 3.70 | 0.89 |
| **Postoperative complications** |  |  |  |  |
| No postoperative complications | 309 | 0.84 | 0.50, 1.42 | 0.52 |
| Anastomosis leak | 309 | 2.16 | 0.78, 5.98 | 0.14 |
| Pelvic sepsis | 309 | 1.0 | 0.14, 7.21 | >0.99 |
| Abdominal abscess requiring drainage | 309 | 0.39 | 0.05, 2.85 | 0.36 |
| Ileus | 309 | 1.09 | 0.59, 2.03 | 0.77 |
| Fistulas or sinus tracts developed until ileostomy takedown | 309 | 2.13 | 0.29, 15.4 | 0.46 |
| **Initial endoscopic phenotype** |  |  |  |  |
| Normal | 317 | 0.25 | 0.09, 0.68 | 0.007 |
| Afferent limb involvement | 222 | 1.66 | 0.72, 3.86 | 0.24 |
| Inlet involvement | 256 | 1.44 | 0.76, 2.73 | 0.27 |
| Diffuse inflammation of the pouch body | 316 | 1.01 | 0.40, 2.54 | 0.98 |
| Focal inflammation of the pouch body | 316 | 2.80 | 1.42, 5.53 | 0.003 |
| Cuffitis | 207 | 1.37 | 0.77, 2.44 | 0.29 |
| Pouch-related fistula | 317 | 0.00 | 0.00, Inf | >0.99 |
| **Number of inflammatory phenotypes at the first scope** | 317 | 1.40 | 1.11, 1.77 | 0.004 |
| ^1^HR = Hazard Ratio, CI = Confidence Interval | | | | |

Table S6. Subgroup Analysis of Patients with Focal Inflammation of the Pouch Body at the Initial Postoperative Pouchoscopy

|  | | **Chronic pouchitis** | |  |
| --- | --- | --- | --- | --- |
| **Variable** | **N** | **No**, N = 145^1^ | **Yes**, N = 50^1^ | **P-value**^2^ |
| **Findings of focal inflammation at the initial scope** |  |  |  |  |
| Normal (TIP) | 195 | 72 (50%) | 23 (46%) | 0.74 |
| Ulceration (TIP) | 195 | 0 (0%) | 1 (2.0%) | 0.26 |
| Erythema/edema (TIP) | 195 | 13 (9.0%) | 3 (6.0%) | 0.77 |
| Erosions/friability (TIP) | 195 | 20 (14%) | 3 (6.0%) | 0.20 |
| Stenosis (TIP) | 195 | 0 (0%) | 0 (0%) |  |
| Granularity (TIP) | 195 | 0 (0%) | 0 (0%) |  |
| Mucous exudate (TIP) | 195 | 0 (0%) | 0 (0%) |  |
| Loss of vascular pattern (TIP) | 195 | 2 (1.4%) | 2 (4.0%) | 0.27 |
| Normal (Proximal pouch) | 195 | 25 (17%) | 11 (22%) | 0.53 |
| Ulceration (Proximal pouch) | 195 | 23 (16%) | 12 (24%) | 0.20 |
| Erythema/edema (Proximal pouch) | 195 | 55 (38%) | 22 (44%) | 0.50 |
| Erosions/friability (Proximal pouch) | 195 | 61 (42%) | 18 (36%) | 0.51 |
| Stenosis (Proximal pouch) | 195 | 0 (0%) | 0 (0%) |  |
| Granularity (Proximal pouch) | 195 | 5 (3.4%) | 0 (0%) | 0.33 |
| Mucous exudate (Proximal pouch) | 195 | 9 (6.2%) | 4 (8.0%) | 0.74 |
| Loss of vascular pattern (Proximal pouch) | 195 | 16 (11%) | 4 (8.0%) | 0.79 |
| Normal (Distal pouch) | 195 | 28 (19%) | 6 (12%) | 0.29 |
| Ulceration (Distal pouch) | 195 | 27 (19%) | 12 (24%) | 0.42 |
| Erythema/edema (Distal pouch) | 195 | 70 (48%) | 32 (64%) | 0.071 |
| Erosions/friability (Distal pouch) | 195 | 58 (40%) | 21 (42%) | 0.87 |
| Stenosis (Distal pouch) | 195 | 1 (0.7%) | 0 (0%) | >0.99 |
| Granularity (Distal pouch) | 195 | 8 (5.5%) | 3 (6.0%) | >0.99 |
| Mucous exudate (Distal pouch) | 195 | 11 (7.6%) | 6 (12%) | 0.38 |
| Loss of vascular pattern (Distal pouch) | 195 | 24 (17%) | 11 (22%) | 0.40 |
|  |  |  |  |  |
| **Initial phenotype** |  |  |  |  |
| Normal | 195 | 0 (0%) | 0 (0%) |  |
| Afferent limb involvement | 195 | 16 (11%) | 4 (8.0%) | 0.79 |
| Inlet involvement | 195 | 27 (19%) | 10 (20%) | 0.84 |
| Diffuse inflammation of the pouch body | 195 | 0 (0%) | 0 (0%) |  |
| Focal inflammation of the pouch body | 195 | 145 (100%) | 50 (100%) |  |
| Cuffitis | 195 | 54 (37%) | 26 (52%) | 0.095 |
| Pouch-related fistula | 195 | 0 (0%) | 0 (0%) |  |
| Number of inflammatory phenotypes at the initial scope | 195 |  |  | 0.36 |
| 1 |  | 69 (48%) | 18 (36%) |  |
| 2-3 |  | 70 (48%) | 30 (60%) |  |
| 4-5 |  | 6 (4.1%) | 2 (4.0%) |  |
|  |  |  |  |  |
| **Overall phenotype** |  |  |  |  |
| Normal (Overall) | 195 | 0 (0%) | 0 (0%) |  |
| Afferent limb involvement (Overall) | 195 | 27 (19%) | 10 (20%) | 0.84 |
| Inlet involvement (Overall) | 195 | 47 (32%) | 22 (44%) | 0.17 |
| Diffuse inflammation of the pouch body (Overall) | 195 | 9 (6.2%) | 15 (30%) | <0.001 |
| Focal inflammation of the pouch body (Overall) | 195 | 136 (94%) | 35 (70%) | <0.001 |
| Cuffitis (Overall) | 195 | 60 (41%) | 34 (68%) | 0.002 |
| Pouch-related fistula (Overall) | 195 | 2 (1.4%) | 7 (14%) | 0.001 |
| Number of inflammatory phenotypes (Overall) | 195 |  |  | 0.005 |
| 1 |  | 57 (39%) | 10 (20%) |  |
| 2-3 |  | 75 (52%) | 28 (56%) |  |
| 4-5 |  | 13 (9.0%) | 12 (24%) |  |
| ^1^Frequency (%) | | | | |
| ^2^Wilcoxon rank sum test; Fisher's exact test | | | | |

**Table S7. Univariate Analysis to Assess Factors Contributing to Chronic Pouchitis**

|  | | **Chronic pouchitis** | |  |  |
| --- | --- | --- | --- | --- | --- |
| **Variable** | **N** | **No**, N = 265^1^ | **Yes**, N = 125^1^ | **P-value**^2^ |  |
| **Age at diagnosis (yrs)** | 388 | 34 (24, 47) | 28 (19, 39) | <0.001 |  |
| **Age at colectomy (yrs)** | 390 | 46 (33, 57) | 39 (25, 49) | <0.001 |  |
| **Disease duration until surgery (yrs)** | 388 | 5 (2, 14) | 5 (2, 11) | 0.27 |  |
| **Body mass index** | 389 | 20.4 (18.5, 22.8) | 20.4 (18.3, 23.0) | 0.94 |  |
| **Gender** | 390 |  |  | 0.23 |  |
| Female |  | 119 (45%) | 48 (38%) |  |  |
| Male |  | 146 (55%) | 77 (62%) |  |  |
| **Montreal classification** | 379 |  |  | 0.72 |  |
| Proctitis |  | 5 (1.9%) | 1 (0.9%) |  |  |
| Left-sided colitis |  | 44 (17%) | 22 (19%) |  |  |
| Extensive colitis |  | 213 (81%) | 94 (80%) |  |  |
| **Primary sclerosing cholangitis** | 389 |  |  | 0.72 |  |
| No |  | 259 (98%) | 122 (98%) |  |  |
| Yes |  | 5 (1.9%) | 3 (2.4%) |  |  |
| **Current smoker** | 285 |  |  | 0.51 |  |
| No |  | 185 (95%) | 89 (98%) |  |  |
| Yes |  | 9 (4.6%) | 2 (2.2%) |  |  |
| **Stage of ileal pouch-anal anastomosis** | 386 |  |  | 0.013 |  |
| 1-stage |  | 28 (11%) | 27 (22%) |  |  |
| 2-stages |  | 163 (62%) | 69 (56%) |  |  |
| 3-stage |  | 72 (27%) | 27 (22%) |  |  |
| **Anastomosis type** | 375 |  |  | 0.001 |  |
| Staple |  | 91 (36%) | 66 (54%) |  |  |
| Hand-sewn |  | 162 (64%) | 56 (46%) |  |  |
| **Preoperative treatments** |  |  |  |  |  |
| Tumor necrosis factor inhibitors | 372 | 75 (30%) | 41 (34%) | 0.40 |  |
| Azathioprine/6-mercaptopurine | 372 | 107 (42%) | 59 (49%) | 0.26 |  |
| Ustekinumab | 372 | 0 (0%) | 0 (0%) |  |  |
| Vedolizumab | 372 | 3 (1.2%) | 1 (0.8%) | >0.99 |  |
| Janus kinase inhibitors | 372 | 8 (3.2%) | 4 (3.3%) | >0.99 |  |
| Oral aminosalicylates | 372 | 218 (87%) | 106 (88%) | 0.74 |  |
| Calcineurin inhibitors | 372 | 75 (30%) | 35 (29%) | >0.99 |  |
| Systemic steroids | 372 | 220 (87%) | 114 (95%) | 0.027 |  |
| Apheresis | 372 | 113 (45%) | 55 (46%) | 0.91 |  |
| No preoperative treatments | 372 | 1 (0.4%) | 1 (0.8%) | 0.54 |  |
| **Indications for colectomy** |  |  |  |  |  |
| Medically refractory | 388 | 172 (65%) | 93 (75%) | 0.061 |  |
| Dysplasia/Colorectal Cancer | 388 | 64 (24%) | 21 (17%) | 0.12 |  |
| Fulminant colitis | 388 | 4 (1.5%) | 2 (1.6%) | >0.99 |  |
| Toxic megacolon | 388 | 9 (3.4%) | 4 (3.2%) | >0.99 |  |
| Massive hemorrhage | 388 | 9 (3.4%) | 5 (4.0%) | 0.77 |  |
| Perforation | 388 | 11 (4.2%) | 4 (3.2%) | 0.78 |  |
| **Postoperative complications** |  |  |  |  |  |
| No postoperative complications | 375 | 155 (60%) | 59 (51%) | 0.14 |  |
| Anastomosis leak | 375 | 12 (4.6%) | 10 (8.7%) | 0.15 |  |
| Pelvic sepsis | 375 | 3 (1.2%) | 3 (2.6%) | 0.38 |  |
| Abdominal abscess requiring drainage | 375 | 17 (6.5%) | 2 (1.7%) | 0.071 |  |
| Ileus | 375 | 58 (22%) | 30 (26%) | 0.43 |  |
| Fistulas or sinus tracts developed until ileostomy takedown | 375 | 3 (1.2%) | 4 (3.5%) | 0.21 |  |
| **Overall endoscopic phenotype** |  |  |  |  |  |
| Normal (Overall) | | 390 | 54 (20%) | 2 (1.6%) | <0.001 |
| Afferent limb involvement (Overall) | 334 | 47 (20%) | 41 (40%) | <0.001 |  |
| Inlet involvement (Overall) | 361 | 87 (35%) | 81 (72%) | <0.001 |  |
| Diffuse inflammation of the pouch body (Overall) | 388 | 46 (17%) | 60 (48%) | <0.001 |  |
| Focal inflammation of the pouch body (Overall) | 388 | 155 (59%) | 60 (48%) | 0.063 |  |
| Cuffitis (Overall) | 263 | 83 (48%) | 72 (79%) | <0.001 |  |
| Pouch-related fistula (Overall) | 390 | 6 (2.3%) | 13 (10%) | 0.001 |  |
| **Number of inflammatory phenotypes (Overall)** | 390 | 2.00 (1.00, 2.00) | 3.00 (2.00, 4.00) | <0.001 |  |
| ^1^Median (IQR) or Frequency (%) | | | | |  |
| ^2^Wilcoxon rank sum test; Fisher's exact test | | | | |  |

**Table S8. Univariate Analysis to Assess Factors Predicting Pouch Failure**

**(Cox Proportional Hazards Model)**

| **Characteristic** | **N** | **HR**^1^ | **95% CI**^1^ | **p-value** |
| --- | --- | --- | --- | --- |
| **Age at diagnosis (yrs)** | 376 | 0.95 | 0.91, 1.00 | 0.041 |
| **Age at colectomy (yrs)** | 376 | 0.95 | 0.91, 0.99 | 0.011 |
| **Disease duration until surgery (yrs)** | 376 | 0.97 | 0.91, 1.04 | 0.41 |
| **Body mass index** | 376 | 0.95 | 0.82, 1.10 | 0.48 |
| **Gender** | 376 |  |  |  |
| Female |  | — | — |  |
| Male |  | 0.69 | 0.25, 1.91 | 0.48 |
| **Montreal classification** | 364 |  |  |  |
| Proctitis |  | — | — |  |
| Left-sided colitis |  | 3,826,936 | 0.00, Inf | >0.99 |
| Extensive colitis |  | 10,662,766 | 0.00, Inf | >0.99 |
| **Primary sclerosing cholangitis** | 375 | 0.00 | 0.00, Inf | >0.99 |
| **Current smoker** | 274 | 0.00 | 0.00, Inf | >0.99 |
| **Stage of ileal pouch-anal anastomosis** | 373 |  |  |  |
| 1-stage |  | — | — |  |
| 2-stages |  | 0.91 | 0.24, 3.47 | 0.89 |
| 3-stage |  | 1.02 | 0.22, 4.64 | 0.98 |
| **Anastomosis type** | 361 |  |  |  |
| Staple |  | — | — |  |
| Hand-sewn |  | 0.95 | 0.34, 2.65 | 0.93 |
| **Preoperative treatments** |  |  |  |  |
| Tumor necrosis factor inhibitors | 365 | 4.17 | 1.42, 12.2 | 0.009 |
| Azathioprine/6-mercaptopurine | 365 | 3.44 | 1.08, 11.0 | 0.037 |
| Vedolizumab | 365 | 0.00 | 0.00, Inf | >0.99 |
| Janus kinase inhibitors | 365 | 0.00 | 0.00, Inf | >0.99 |
| Oral aminosalicylates | 365 | 2.12 | 0.28, 16.2 | 0.47 |
| Calcineurin inhibitors | 365 | 2.67 | 0.93, 7.62 | 0.067 |
| Systemic steroids | 365 | 1.24 | 0.16, 9.50 | 0.84 |
| Apheresis | 365 | 1.95 | 0.65, 5.82 | 0.23 |
| No preoperative treatments | 365 | 0.00 | 0.00, Inf | >0.99 |
| **Indications for colectomy** |  |  |  |  |
| Medically refractory | 374 | 1.57 | 0.44, 5.58 | 0.49 |
| Dysplasia/Colorectal Cancer | 374 | 0.61 | 0.14, 2.73 | 0.52 |
| Fulminant colitis | 374 | 0.00 | 0.00, Inf | >0.99 |
| Toxic megacolon | 374 | 0.00 | 0.00, Inf | >0.99 |
| Massive hemorrhage | 374 | 1.75 | 0.23, 13.3 | 0.59 |
| Perforation | 374 | 0.00 | 0.00, Inf | >0.99 |
| **Postoperative complications** |  |  |  |  |
| No postoperative complications | 361 | 0.26 | 0.08, 0.80 | 0.019 |
| Anastomosis leak | 361 | 9.65 | 3.06, 30.5 | <0.001 |
| Pelvic sepsis | 361 | 0.00 | 0.00, Inf | >0.99 |
| Abdominal abscess requiring drainage | 361 | 0.00 | 0.00, Inf | >0.99 |
| Ileus | 361 | 1.68 | 0.57, 4.92 | 0.34 |
| Fistulas or sinus tracts developed until ileostomy takedown | 361 | 10.2 | 2.28, 45.8 | 0.002 |
| **Initial endoscopic phenotype** |  |  |  |  |
| Normal | 376 | 0.00 | 0.00, Inf | >0.99 |
| Afferent limb involvement | 266 | 2.19 | 0.55, 8.74 | 0.27 |
| Inlet involvement | 310 | 8.73 | 1.89, 40.4 | 0.006 |
| Diffuse inflammation of the pouch body | 374 | 0.91 | 0.20, 4.03 | 0.90 |
| Focal inflammation of the pouch body | 374 | 1.67 | 0.53, 5.25 | 0.38 |
| Cuffitis | 240 | 0.81 | 0.26, 2.55 | 0.72 |
| Pouch-related fistula | 376 | 0.00 | 0.00, Inf | >0.99 |
| **Number of inflammatory phenotypes at the first scope** | 376 | 1.55 | 1.00, 2.42 | 0.053 |
| ^1^HR = Hazard Ratio, CI = Confidence Interval | | | | |

**Table S9. Subgroup Analysis of Patients with Inlet Involvement at the Initial Postoperative Pouchoscopy**

|  | | **Pouch failure** | |  |
| --- | --- | --- | --- | --- |
| **Variable** | **N** | **No**, N = 96^1^ | **Yes**, N = 9^1^ | **P-value**^2^ |
| **Findings of inlet involvement at the initial scope** |  |  |  |  |
| Normal (Inlet) | 105 | 0 (0%) | 0 (0%) |  |
| Ulceration (Inlet) | 105 | 23 (24%) | 5 (56%) | 0.055 |
| Erythema/edema (Inlet) | 105 | 65 (68%) | 7 (78%) | 0.72 |
| Erosions/friability (Inlet) | 105 | 55 (57%) | 6 (67%) | 0.73 |
| Stenosis (Inlet) | 105 | 1 (1.0%) | 0 (0%) | >0.99 |
| Granularity (Inlet) | 105 | 24 (25%) | 1 (11%) | 0.68 |
| Mucous exudate (Inlet) | 105 | 21 (22%) | 1 (11%) | 0.68 |
| Loss of vascular pattern (Inlet) | 105 | 30 (31%) | 4 (44%) | 0.47 |
|  |  |  |  |  |
| **Initial phenotype** |  |  |  |  |
| Normal | 105 | 0 (0%) | 0 (0%) |  |
| Afferent limb involvement | 105 | 43 (45%) | 3 (33%) | 0.73 |
| Inlet involvement | 105 | 96 (100%) | 9 (100%) |  |
| Diffuse inflammation of the pouch body | 105 | 49 (51%) | 2 (22%) | 0.16 |
| Focal inflammation of the pouch body | 105 | 43 (45%) | 6 (67%) | 0.30 |
| Cuffitis | 105 | 33 (34%) | 2 (22%) | 0.71 |
| Pouch-related fistula | 105 | 0 (0%) | 0 (0%) |  |
| Number of inflammatory phenotypes at the initial scope | 105 |  |  | >0.99 |
| 1 |  | 4 (4.2%) | 0 (0%) |  |
| 2-3 |  | 74 (77%) | 8 (89%) |  |
| 4-5 |  | 18 (19%) | 1 (11%) |  |
|  |  |  |  |  |
| **Overall phenotype** |  |  |  |  |
| Normal (Overall) | 105 | 0 (0%) | 0 (0%) |  |
| Afferent limb involvement (Overall) | 105 | 47 (49%) | 6 (67%) | 0.49 |
| Inlet involvement (Overall) | 105 | 96 (100%) | 9 (100%) |  |
| Diffuse inflammation of the pouch body (Overall) | 105 | 60 (63%) | 5 (56%) | 0.73 |
| Focal inflammation of the pouch body (Overall) | 105 | 33 (34%) | 4 (44%) | 0.72 |
| Cuffitis (Overall) | 105 | 37 (39%) | 6 (67%) | 0.16 |
| Pouch-related fistula (Overall) | 105 | 1 (1.0%) | 5 (56%) | <0.001 |
| Number of inflammatory phenotypes (Overall) | 105 |  |  | 0.008 |
| 1 |  | 3 (3.1%) | 0 (0%) |  |
| 2-3 |  | 69 (72%) | 2 (22%) |  |
| 4-5 |  | 24 (25%) | 7 (78%) |  |
| ^1^Frequency (%) | | | | |
| ^2^Wilcoxon rank sum test; Fisher's exact test | | | | |

**Table S10. Univariate Analysis to Assess Factors Contributing to Pouch Failure**

|  | | **Pouch failure** | |  |
| --- | --- | --- | --- | --- |
| **Variable** | **N** | **No**, N = 370^1^ | **Yes**, N = 19^1^ | **P-value**^2^ |
| **Age at diagnosis (yrs)** | 387 | 32 (22, 45) | 26 (19, 34) | 0.034 |
| **Age at colectomy (yrs)** | 389 | 44 (31, 55) | 34 (22, 43) | 0.008 |
| **Disease duration until surgery (yrs)** | 387 | 5 (2, 14) | 4 (2, 10) | 0.56 |
| **Body mass index** | 388 | 20.5 (18.4, 22.9) | 19.5 (17.5, 23.0) | 0.24 |
| **Gender** | 389 |  |  | 0.81 |
| Female |  | 158 (43%) | 9 (47%) |  |
| Male |  | 212 (57%) | 10 (53%) |  |
| **Montreal classification** | 377 |  |  | 0.42 |
| Proctitis |  | 6 (1.7%) | 0 (0%) |  |
| Left-sided colitis |  | 64 (18%) | 1 (5.6%) |  |
| Extensive colitis |  | 289 (81%) | 17 (94%) |  |
| **Primary sclerosing cholangitis** | 388 |  |  | >0.99 |
| No |  | 361 (98%) | 19 (100%) |  |
| Yes |  | 8 (2.2%) | 0 (0%) |  |
| **Current smoker** | 284 |  |  | >0.99 |
| No |  | 257 (96%) | 16 (100%) |  |
| Yes |  | 11 (4.1%) | 0 (0%) |  |
| **Stage of ileal pouch-anal anastomosis** | 384 |  |  | 0.89 |
| 1-stage |  | 52 (14%) | 3 (17%) |  |
| 2-stages |  | 219 (60%) | 11 (61%) |  |
| 3-stage |  | 95 (26%) | 4 (22%) |  |
| **Anastomosis type** | 374 |  |  | >0.99 |
| Staple |  | 148 (42%) | 8 (42%) |  |
| Hand-sewn |  | 207 (58%) | 11 (58%) |  |
| **Preoperative treatments** |  |  |  |  |
| Tumor necrosis factor inhibitors | 371 | 105 (30%) | 10 (59%) | 0.016 |
| Azathioprine/6-mercaptopurine | 371 | 153 (43%) | 11 (65%) | 0.13 |
| Ustekinumab | 371 | 0 (0%) | 0 (0%) |  |
| Vedolizumab | 371 | 4 (1.1%) | 0 (0%) | >0.99 |
| Janus kinase inhibitors | 371 | 11 (3.1%) | 1 (5.9%) | 0.44 |
| Oral aminosalicylates | 371 | 307 (87%) | 15 (88%) | >0.99 |
| Calcineurin inhibitors | 371 | 100 (28%) | 9 (53%) | 0.052 |
| Systemic steroids | 371 | 317 (90%) | 16 (94%) | >0.99 |
| Apheresis | 371 | 156 (44%) | 10 (59%) | 0.32 |
| No preoperative treatments | 371 | 2 (0.6%) | 0 (0%) | >0.99 |
| **Indications for colectomy** |  |  |  |  |
| Medically refractory | 387 | 248 (67%) | 16 (84%) | 0.20 |
| Dysplasia/Colorectal Cancer | 387 | 83 (23%) | 2 (11%) | 0.27 |
| Fulminant colitis | 387 | 6 (1.6%) | 0 (0%) | >0.99 |
| Toxic megacolon | 387 | 13 (3.5%) | 0 (0%) | >0.99 |
| Massive hemorrhage | 387 | 13 (3.5%) | 1 (5.3%) | 0.51 |
| Perforation | 387 | 15 (4.1%) | 0 (0%) | >0.99 |
| **Postoperative complications** |  |  |  |  |
| No postoperative complications | 374 | 207 (58%) | 6 (32%) | 0.031 |
| Anastomosis leak | 374 | 17 (4.8%) | 5 (26%) | 0.003 |
| Pelvic sepsis | 374 | 6 (1.7%) | 0 (0%) | >0.99 |
| Abdominal abscess requiring drainage | 374 | 19 (5.4%) | 0 (0%) | 0.61 |
| Ileus | 374 | 82 (23%) | 6 (32%) | 0.41 |
| Fistulas or sinus tracts developed until ileostomy takedown | 374 | 5 (1.4%) | 2 (11%) | 0.044 |
| **Overall endoscopic phenotype** |  |  |  |  |
| Normal (Overall) | 389 | 56 (15%) | 0 (0%) | 0.089 |
| Afferent limb involvement (Overall) | 333 | 78 (25%) | 10 (67%) | <0.001 |
| Inlet involvement (Overall) | 360 | 152 (44%) | 14 (78%) | 0.007 |
| Diffuse inflammation of the pouch body (Overall) | 387 | 98 (27%) | 7 (37%) | 0.43 |
| Focal inflammation of the pouch body (Overall) | 387 | 203 (55%) | 12 (63%) | 0.64 |
| Cuffitis (Overall) | 263 | 142 (57%) | 13 (81%) | 0.070 |
| Pouch-related fistula (Overall) | 389 | 12 (3.2%) | 7 (37%) | <0.001 |
| **Number of inflammatory phenotypes (Overall)** | 389 | 2.00 (1.00, 3.00) | 4.00 (2.00, 4.00) | <0.001 |
| ^1^Median (IQR) or Frequency (%) | | | | |
| ^2^Wilcoxon rank sum test; Fisher's exact test | | | | |

**Table S11. Univariate Analysis to Assess Factors Contributing to Acute Pouchitis**

|  | | **Acute pouchitis** | |  |  |
| --- | --- | --- | --- | --- | --- |
| **Variable** | **N** | **No**, N = 262^1^ | **Yes**, N = 115^1^ | **P-value**^2^ |  |
| **Age at diagnosis (yrs)** | 375 | 33 (22, 46) | 29 (21, 39) | 0.067 |  |
| **Age at colectomy (yrs)** | 377 | 45 (32, 55) | 39 (31, 52) | 0.044 |  |
| **Disease duration until surgery (yrs)** | 375 | 6 (2, 13) | 5 (2, 14) | 0.79 |  |
| **Body mass index** | 377 | 20.5 (18.4, 23.0) | 20.4 (18.4, 22.8) | 0.81 |  |
| **Gender** | 377 |  |  | 0.37 |  |
| Female |  | 116 (44%) | 45 (39%) |  |  |
| Male |  | 146 (56%) | 70 (61%) |  |  |
| **Montreal classification** | 367 |  |  | 0.89 |  |
| Proctitis |  | 5 (1.9%) | 1 (0.9%) |  |  |
| Left-sided colitis |  | 44 (17%) | 18 (16%) |  |  |
| Extensive colitis |  | 208 (81%) | 91 (83%) |  |  |
| **Primary sclerosing cholangitis** | 376 |  |  | >0.99 |  |
| No |  | 257 (98%) | 112 (98%) |  |  |
| Yes |  | 5 (1.9%) | 2 (1.8%) |  |  |
| **Current smoker** | 277 |  |  | 0.52 |  |
| No |  | 187 (95%) | 79 (98%) |  |  |
| Yes |  | 9 (4.6%) | 2 (2.5%) |  |  |
| **Stage of ileal pouch-anal anastomosis** | 374 |  |  | 0.12 |  |
| 1-stage |  | 40 (15%) | 10 (8.7%) |  |  |
| 2-stages |  | 157 (61%) | 69 (60%) |  |  |
| 3-stage |  | 62 (24%) | 36 (31%) |  |  |
| **Anastomosis type** | 363 |  |  | <0.001 |  |
| Staple |  | 130 (51%) | 21 (20%) |  |  |
| Hand-sewn |  | 126 (49%) | 86 (80%) |  |  |
| **Preoperative treatments** |  |  |  |  |  |
| Tumor necrosis factor inhibitors | 362 | 82 (33%) | 27 (24%) | 0.11 |  |
| Azathioprine/6-mercaptopurine | 362 | 114 (46%) | 45 (40%) | 0.36 |  |
| Ustekinumab | 362 | 0 (0%) | 0 (0%) |  |  |
| Vedolizumab | 362 | 3 (1.2%) | 1 (0.9%) | >0.99 |  |
| Janus kinase inhibitors | 362 | 6 (2.4%) | 5 (4.5%) | 0.33 |  |
| Oral aminosalicylates | 362 | 224 (90%) | 90 (80%) | 0.019 |  |
| Calcineurin inhibitors | 362 | 75 (30%) | 31 (28%) | 0.71 |  |
| Systemic steroids | 362 | 220 (88%) | 105 (94%) | 0.13 |  |
| Apheresis | 362 | 119 (48%) | 43 (38%) | 0.11 |  |
| No preoperative treatments | 362 | 1 (0.4%) | 1 (0.9%) | 0.52 |  |
| **Indications for colectomy** |  |  |  |  |  |
| Medically refractory | 376 | 171 (66%) | 85 (74%) | 0.12 |  |
| Dysplasia/Colorectal Cancer | 376 | 61 (23%) | 22 (19%) | 0.42 |  |
| Fulminant colitis | 376 | 4 (1.5%) | 1 (0.9%) | >0.99 |  |
| Toxic megacolon | 376 | 6 (2.3%) | 7 (6.1%) | 0.073 |  |
| Massive hemorrhage | 376 | 10 (3.8%) | 4 (3.5%) | >0.99 |  |
| Perforation | 376 | 12 (4.6%) | 3 (2.6%) | 0.57 |  |
| **Postoperative complications** |  |  |  |  |  |
| No postoperative complications | 364 | 138 (55%) | 72 (64%) | 0.11 |  |
| Anastomosis leak | 364 | 17 (6.7%) | 4 (3.6%) | 0.33 |  |
| Pelvic sepsis | 364 | 4 (1.6%) | 2 (1.8%) | >0.99 |  |
| Abdominal abscess requiring drainage | 364 | 15 (6.0%) | 3 (2.7%) | 0.29 |  |
| Ileus | 364 | 60 (24%) | 25 (22%) | 0.79 |  |
| Fistulas or sinus tracts developed until ileostomy takedown | 364 | 4 (1.6%) | 1 (0.9%) | >0.99 |  |
| **Overall endoscopic phenotype** |  |  |  |  |  |
| Normal (Overall) | | 377 | 55 (21%) | 1 (0.9%) | <0.001 |
| Afferent limb involvement (Overall) | 322 | 47 (22%) | 39 (38%) | 0.003 |  |
| Inlet involvement (Overall) | 349 | 82 (34%) | 76 (68%) | <0.001 |  |
| Diffuse inflammation of the pouch body (Overall) | 375 | 51 (20%) | 48 (42%) | <0.001 |  |
| Focal inflammation of the pouch body (Overall) | 375 | 144 (55%) | 65 (57%) | 0.91 |  |
| Cuffitis (Overall) | 258 | 113 (56%) | 37 (66%) | 0.22 |  |
| Pouch-related fistula (Overall) | 377 | 13 (5.0%) | 5 (4.3%) | >0.99 |  |
| **Number of inflammatory phenotypes (Overall)** | 377 | 2.00 (1.00, 3.00) | 2.00 (2.00, 3.00) | <0.001 |  |
| ^1^Median (IQR) or Frequency (%) | | | | |  |
| ^2^Wilcoxon rank sum test; Fisher's exact test | | | | |  |

Table S12. Multivariate Analysis to Assess Factors Contributing to Acute Pouchitis

| **Characteristic** | **OR**^1^ | **95% CI**^1^ | **p-value*** |
| --- | --- | --- | --- |
| ***Acute pouchitis*** |  |  |  |
| Age at colectomy (yrs) | 0.98781 | 0.96846, 1.00717 | 0.21824 |
| Anastomosis type |  |  |  |
| Staple | — | — |  |
| Hand-sewn | 4.22064 | 2.29086, 8.12107 | **0.00001** |
| Preoperative oral aminosalicylates | 1.23604 | 0.55523, 2.83946 | 0.60912 |
| Toxic megacolon | 2.80157 | 0.75959, 10.7861 | 0.12288 |
| Afferent limb involvement (Overall) | 0.69875 | 0.32684, 1.46307 | 0.34685 |
| Inlet involvement (Overall) | 4.09466 | 1.98006, 8.63416 | **0.00017** |
| Diffuse inflammation of the pouch body (Overall) | 1.68688 | 0.82642, 3.43982 | 0.14924 |
| ^1^OR = Odds Ratio, CI = Confidence Interval  *****The bold values indicate *p-*value <0.05 | | | |
